# Machine Learning For Risk Prediction After Heart Failure Emergency Department Visit or Hospital Admission Using Administrative Health Data

**DOI:** 10.1371/journal.pdig.0000636

**Published:** 2024-10-25

**Authors:** Nowell M. Fine, Sunil V. Kalmady, Weijie Sun, Russ Greiner, Jonathan G. Howlett, James A. White, Finlay A. McAlister, Justin A. Ezekowitz, Padma Kaul

**Affiliations:** 1 Division of Cardiology, Department of Cardiac Sciences, Libin Cardiovascular Institute, Cumming School of Medicine, University of Calgary, Calgary Alberta; 2 Canadian Vigour Center, Katz Group Center for Pharmacy and Health Research, University of Alberta, Edmonton Alberta; 3 Alberta Machine Intelligence Institute, Edmonton Alberta; 4 Department of Computing Sciences, University of Alberta; 5 Stephenson Cardiac Imaging Center, Calgary Alberta; 6 Division of Internal Medicine, University of Alberta, Edmonton Alberta; 7 Division of Cardiology, Department of Medicine, University of Alberta, Edmonton Alberta; Brigham and Women’s Hospital, UNITED STATES OF AMERICA

## Abstract

**Aims:**

Patients visiting the emergency department (ED) or hospitalized for heart failure (HF) are at increased risk for subsequent adverse outcomes, however effective risk stratification remains challenging. We utilized a machine-learning (ML)-based approach to identify HF patients at risk of adverse outcomes after an ED visit or hospitalization using a large regional administrative healthcare data system.

**Methods and results:**

Patients visiting the ED or hospitalized with HF between 2002–2016 in Alberta, Canada were included. Outcomes of interest were 30-day and 1-year HF-related ED visits, HF hospital readmission or all-cause mortality. We applied a feature extraction method using deep feature synthesis from multiple sources of health data and compared performance of a gradient boosting algorithm (CatBoost) with logistic regression modelling. The area under receiver operating characteristic curve (AUC-ROC) was used to assess model performance. We included 50,630 patients with 93,552 HF ED visits/hospitalizations. At 30-day follow-up in the holdout validation cohort, the AUC-ROC for the combined endpoint of HF ED visit, HF hospital readmission or death for the Catboost and logistic regression models was 74.16 (73.18–75.11) versus 62.25 (61.25–63.18), respectively. At 1-year follow-up corresponding values were 76.80 (76.1–77.47) versus 69.52 (68.77–70.26), respectively. AUC-ROC values for the endpoint of all-cause death alone at 30-days and 1-year follow-up were 83.21 (81.83–84.41) versus 69.53 (67.98–71.18), and 85.73 (85.14–86.29) versus 69.40 (68.57–70.26), for the CatBoost and logistic regression models, respectively.

**Conclusions:**

ML-based modelling with deep feature synthesis provided superior risk stratification for HF patients at 30-days and 1-year follow-up after an ED visit or hospitalization using data from a large administrative regional healthcare system.

## Author summary

Patients visiting the emergency department (ED) or hospitalized for heart failure (HF) are at increased risk for subsequent adverse outcomes, however effective risk stratification remains challenging. Healthcare administrative or claims databases capture data on large, unselected cohorts of patients and can be used to monitor HF outcomes at a population-level. The primary objective of our study was to develop and validate a risk prediction ML model for 30-day and 1-year HF ED visits, hospital readmissions or death using administrative health data and evaluate its performance and potential utility from a large regional healthcare system. We used deep feature synthesis for automated feature engineering to pool patient information from multiple data sources and developed models using a gradient boosting-based ML algorithm (CatBoost). From a database that included over 50,000 HF patients, Catboost demonstrated superior prognostic utility and precision to both conventional logistic regression and other ML-based risk prediction modelling approaches for all study endpoints. This model may be of value to healthcare administrators for developing strategies to reduce adverse outcomes for hospitalized HF patients.

## Introduction

Heart failure (HF) remains an important cause of morbidity, mortality and healthcare expenditures world-wide [1–3]. Despite significant advances in therapy over the last several years, emergency department (ED) visits and hospital admissions for HF remain high and represent an important burden on patients and healthcare systems [4,5]. Identifying patients with HF at high risk for future ED visits and rehospitalization remains a high priority to develop potential prevention strategies. However, prior studies that have endeavoured to risk stratify which patients are at greatest risk for rehospitalization have reported mixed results. [6,7].

Healthcare administrative or claims databases capture data on large, unselected cohorts of patients and have been used extensively to monitor HF readmission and mortality outcomes at a population-level [8,9]. Often the variables used are restricted to conventional demographic and historical patient characteristics such as age, sex, key comorbidities and historical counts of clinical visits, among others [10]. More recently, there has been a move towards investigating the use of new approaches, including machine learning (ML) based techniques [6,11,12]. However, a majority of these studies are based on data from clinical records, imaging, or laboratory data for relatively small cohorts of patients captured at single centres or in structured clinical trials. Alternatively, administrative health data routinely generated through use of the electronic health records can be employed, however, to date have focused on HF hospitalization and mortality outcomes alone and have not considered HF-related ED visits [9]. Further, the exploration of feature engineering approaches based on ‘deep feature synthesis’ may provide unique value [13], applying transformation and aggregation of variables into novel features. This may be particularly useful for application to administrative health datasets that inherently capture complex and temporally structured information in relational databases.

The primary objective of our study was to develop and validate a risk prediction ML model for 30-day and 1-year HF ED visits, hospital readmissions or death using administrative health data and evaluate its performance and potential utility to healthcare administrators at a population level. To perform this objective, we used administrative health data from a large regional healthcare system among patients presenting to the ED or hospital with HF. We used deep feature synthesis for automated feature engineering to pool patient information from multiple data sources and developed models using a gradient boosting-based ML algorithm.

## Methods

### Data sources

The province of Alberta, Canada has a single-payer, government funded healthcare system that provides universal access to over 4.4 million people for hospital, ED, and physician services. We used de-identified data from administrative databases maintained by the regional healthcare authority, Alberta Health, for the period of 2002–2016, including (1) the Discharge Abstract Database (DAD), which records the admission date, discharge date, most responsible diagnosis, and up to 24 other diagnoses, most responsible intervention, and up to 19 other interventions, special care units and physician specialities for all acute care hospitalizations; (2) the National Ambulatory Care Reporting System (NACRS) database, which records all patient visits to hospital-based physicians’ offices or EDs; (3) the Practitioner Claims Database, which tracks all physician claims for healthcare services; (4) the Alberta Health Care Insurance Plan Registry (AHCIP), which provides demographic information including sex, socio-economic status, residence, urbanicity and ethnicity; and (5) mortality data obtained from the AHCIP and vital status death registry. This study received ethics approval from the Health Ethics Research Board at the University of Alberta and the requirement for informed written consent of patients included was waived. This study conforms with the principles outlined in the Declaration of Helsinki.

### Analysis cohort

**Fig 1:**
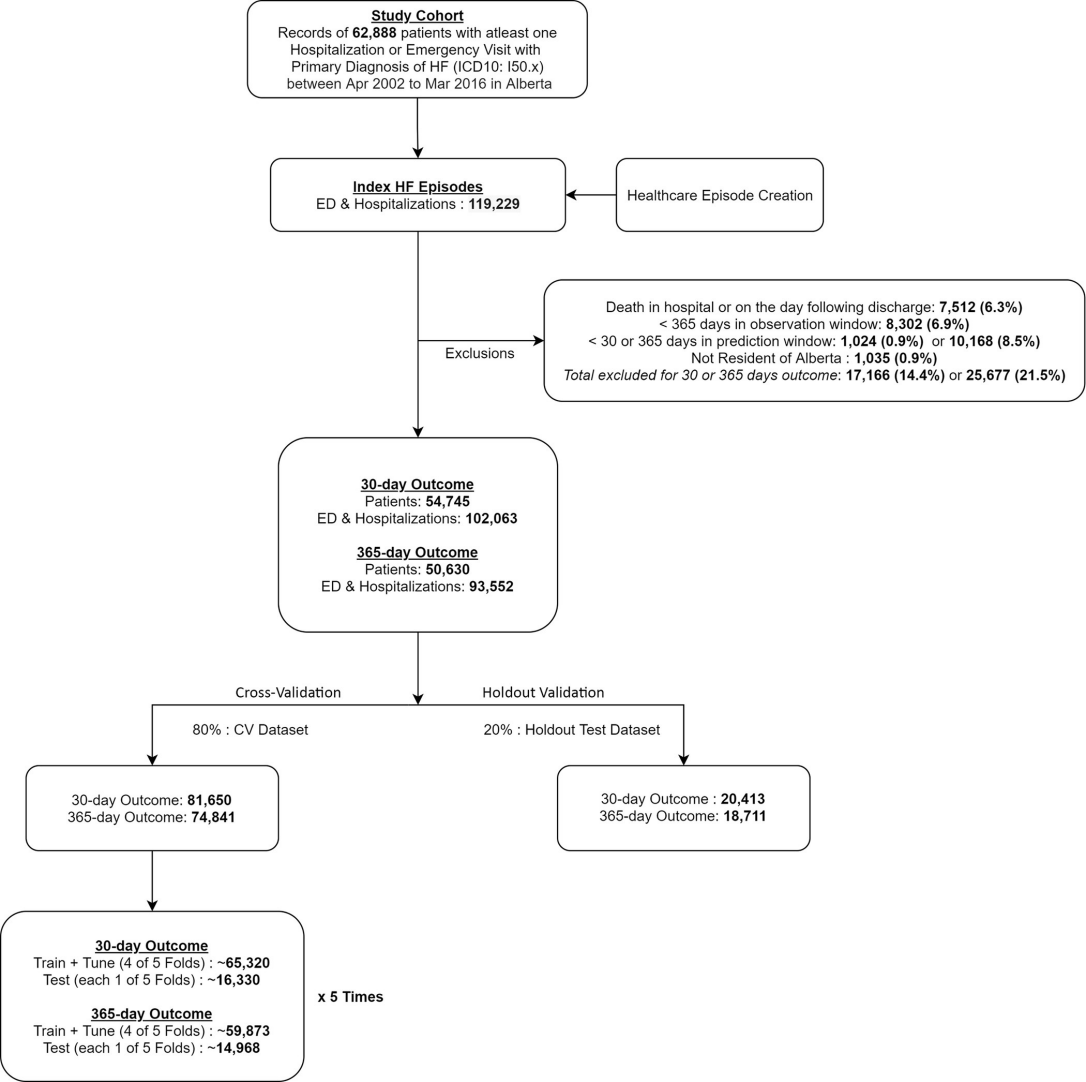
Flowchart describing samples sizes (index events) in the analysis cohort, used for training and for validating the models for each outcome, after applying the episode creation and study exclusion criteria.

**Fig 2:**
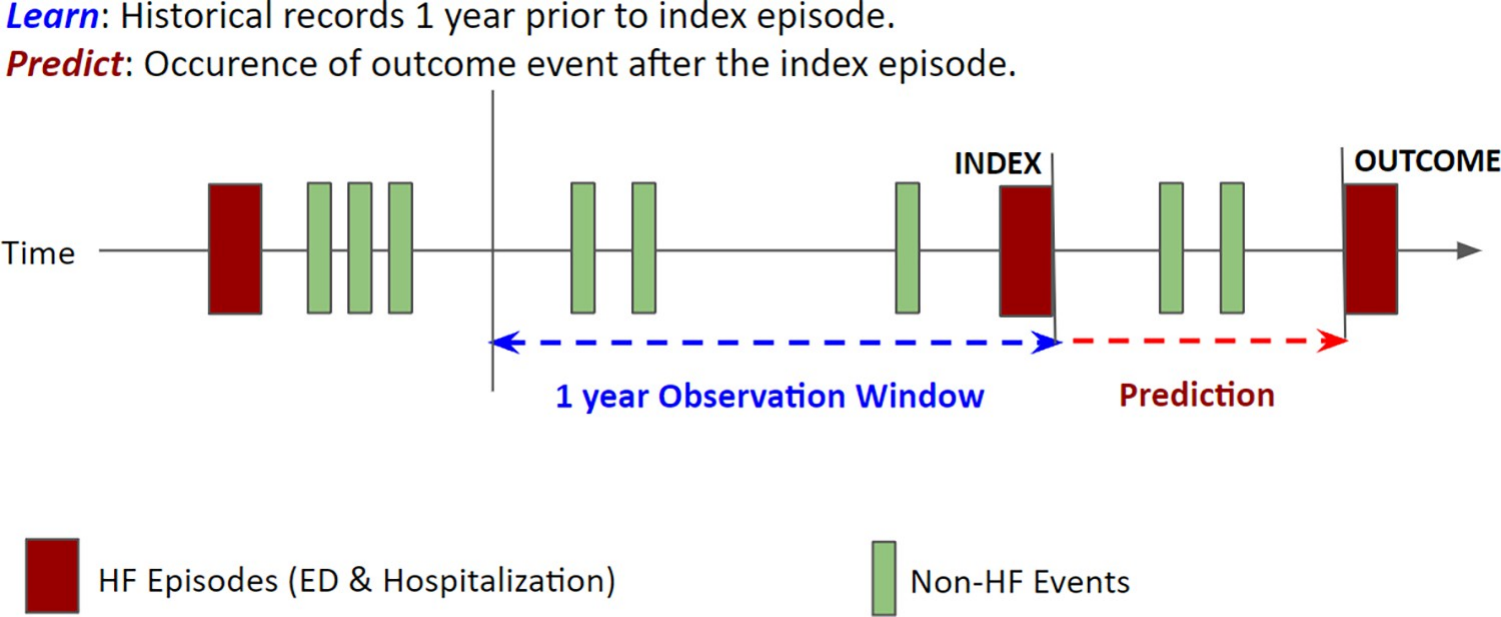
Schematic of observation window and prediction tasks employed in the study. ED-emergency department visit, HF-heart failure.

All ED visits or hospitalizations with a primary diagnosis of HF (ICD10 code starting with I50) between the period of April 1, 2002, to March 31, 2016, were considered index events and used as a unit of analysis in our prediction tasks, including subsequent HF ED visits and hospital readmissions (Fig 1). Any patient with at least one HF index event during this time-period was included in the study cohort. Patients who were not Alberta residents (or without population registry record) were excluded. Furthermore, the following events were excluded from being assigned as index events: a) death during the same hospital visit or on the day following discharge (as predicting in-hospital death was not an objective of this analysis), b) less than 1-year in the observation window (events occurring prior to April 1, 2002), c) less than 30 days or 1-year in the prediction window (events occurring after February 29, 2016, or March 31, 2015, for 30-day and 1-year outcomes, respectively). Because certain healthcare events may be associated with multiple consecutive recorded events or visits within a short-time period (for example, ED to hospital ward or hospital to hospital transfers for HF hospitalization), we grouped these events into a single episode based on the time-period between admissions (within 48 hours), discharge destination code (whether the patient is discharged home or elsewhere) and hospital/facility identifier code (whether patient is transferred from one hospital to another). The flowchart of the decision tree used for episode definition is outlined in S1 Fig. For our observation window, we used a 1-year of lookback period prior to the discharge date of the index event (Fig 2). We collected the variables available during this period to construct subsequent model input features.

### Prediction tasks and endpoints

Prediction tasks were developed to predict the endpoint of HF ED visit, hospital readmission or all-cause death within 30 days and 1-year from the date of discharge from an index HF event (for example, if start date of k+1th HF event minus discharge date of kth HF event is less than 30 days or 1-year, respectively) (Fig 2). The composite endpoint of HF ED visit, HF hospital readmission, or death was used for the primary analysis. We also developed models to predict the occurrence of all-cause death alone within 30 days and for within 1-year of discharge date. Fig 1 provides a study flowchart describing samples sizes (index events) used for training and for validating the models for each outcome, after applying the episode creation and study exclusion criteria. A secondary analysis was conducted for the outcome of HF hospital readmission or all-cause death (not including the endpoint of HF ED visits). S2 Fig provides a corresponding study flowchart describing samples sizes (index events) used for training and for validating the models for each outcome for the secondary analysis.

### Learning algorithms

We employed two learning algorithms for this analysis, CatBoost and logistic regression. CatBoost is a gradient boosting algorithm that was selected because it can learn directly from categorical features (and many clinical features are categorical in nature) without the need for encoding and can effectively process missing features at training or performance time without the need for additional preprocessing [14]. In comparison to this method, we also employed L2-regularized logistic regression with one-hot encoded categorical features, as logistic regression is a frequently used method developing clinical risk prediction models and its ease of interpretability. We used binary cross-entropy (also known as Log Loss) as an objective function and ‘early stopping’ procedure to avoid over-fitting. The models were implemented using Numpy (version 1.19), Pandas (version 1.1), Featuretools (version 0.17), scikit-learn (version 0.24), scipy (version 1.5) and CatBoost (version 0.24) in Python (version 3.9), with use of default training parameters except where specified. We trained all our models on a high-performance platform with 8 available Tesla V100GPUs and 32GB of RAM per GPU.

### Training and testing cohorts

We evaluated our models using cross-validation and holdout validation methods using randomized data splits (Fig 1). For cross-validation, we used five-fold cross-validation within 80% of the whole dataset (CV dataset, 5 learned models M1-M5 for each index and outcome combination). Here 80% of the CV dataset was used for training, and the remaining 20% was used for testing. Five models, namely M1-M5, were evaluated with unique 20% test sets. For holdout validation, we trained on the whole of the CV dataset (Model M0) and then tested on the 20% of the whole dataset which was initially held as a holdout set (not seen by the trained model). In all the above scenarios, we used 20% of the training data as a tuning set to track the AUC performance and stopped the training process when there was no further improvement [15].

### Variables and feature design

We used variables from the administrative records of hospitalizations (DAD), ambulatory and emergency visits (NACRS), practitioner billing (Claims database) and demographics (AHCIP). For instance, a particular patient can have multiple hospitalizations, emergency department (ED) visits and physician clinic visits on different dates, all stored as separate tables and each of these encounters can have multiple diagnoses or procedures (S3 Fig). Our variables consisted of mixed data types: numeric, categorical or Boolean. The full visit variables and their groupings are presented in S1 Table. We employed automatic construction of features from relational data sets via deep feature synthesis (https://www.featuretools.com/). The algorithm follows relationships in the data to a base field, and then sequentially applies mathematical functions along that path to create the final feature [16]. We created custom variables including occurrences / counts of common diagnostic and intervention codes (with at least 0.1% prevalence), number of days since previous visit, and whether the visit date was on a weekend or not. Then, we used the following mathematical operations for aggregation of each variable over multiple historical visits during the observation window, as appropriate to the data type: count, percentage true, median, maximum, standard deviation, number of unique categories, mode, second and third most common category, entropy, last record and linear trend with visit dates. This generated a total of 6,475 features for each training instance (index event). This set was then reduced to 2,226 after removing features with a significant proportion of missing values (>35% missing) and highly correlated features (with correlation coefficient >0.5) for comparison with logistic regression modelling. CatBoost algorithm can handle the missing values directly, whereas for the logistic regression models, the missing values were imputed based on the mean of columns in the training set.

### Evaluation metrics

We evaluated our models with area under receiver operating characteristic curve (AUC-ROC or C-index), a measure of discrimination, and area under precision recall curve (AUC-PRC). A PRC shows the precision or positive predictive power (the probability that an event is observed given a predicted occurrence) as a function of recall or sensitivity (the probability that the model predicts an event among patients with an observed event). AUC-PRC is a metric useful for assessment of classification performance for unbalanced binary responses, and varies on a scale from zero to one, with random performance equal to prevalence of events in the dataset. We compared model performances using the independent sample t-test for the cross-validation, and additionally using the DeLong test for AUC-ROC in the final holdout validation. We also report accuracy, recall (sensitivity), specificity, and precision (positive predictive value). Lastly, we report calibration plots which measure whether predicted probabilities agree with observed proportions and provided CatBoost model performances on subgroups of patients with different underlying HF etiologies and concomitant diagnoses in our holdout test set.

### Model performance and bias analysis based on feature subgroups and temporal trends

We also evaluated the performances of models learned only with specific subgroups of features, namely diagnoses/comorbidities, interventions/medical procedures, demographics, hospital/clinic visits, discharge summary and administration related variables. Further, temporal trends in medical practice and administration of the healthcare system such as policy changes over years can influence the performance of prediction models. To investigate this potential bias, we provide a separate evaluation scheme where holdout validation is performed on the HF episodes from fiscal years 2013 to 2016, that was temporally disjoint from the training set of HF episodes from 2004 to 2016. These results are available publicly on the dashboard: https://cvc-hf-readm-perf-22-0ea970d9e2b0.herokuapp.com/. The codes used to train the models used in this analysis are also available publicly at the following link: https://github.com/jeremykid/Heart-Failure-Readmission-Prediction/tree/main. Lastly, as an ancillary analysis, we also compared CatBoost to the following modeling approaches using the same metrics as described above: 1) deep neural network with four densely connected hidden layers with 330, 433, 511, 449 units for each of the four hidden layers as described by Bat-Erdene et al [17], 2) random forest [18], and 3) support vector machine modelling [19] using default hyperparameters.

## Results

### Study cohort and outcomes

A total of 62,888 patients had at least one ED visit or hospitalization with a primary diagnosis of HF between April 1, 2002, and March 31, 2016 (Fig 1). They accounted for a total of 119,229 ED and hospitalization events and 66,795 hospitalizations alone during the study time-period. After excluding non-residents, patients who died at index, and those with incomplete follow-up, our study included 54,745 patients with 102,063 HF ED visits and hospital readmission events with complete 30-day follow-up and 50,630 patients with 93,552 HF ED visits and hospital readmission events with complete 1-year follow-up (Fig 2). Baseline characteristics of the patients included in the study cohort and the frequency of study endpoints at 30 days and 1-year for study patients are provided in Table 1. At 30-days follow-up, 8.2% of patients had a HF ED visit, an additional 4.8% were readmitted to hospital, while 5.7% died, while corresponding values at 1-year follow-up were 14.8%, 9.5% and 29.1%, respectively. S4 Fig provides the distribution of important study outcomes by year.

**Table 1:** Baseline characteristics and outcomes.

Description	Value
**Demographic**	
Number of episodes	102063
Number of patients	54745
Female Sex	26784 (48.93%)
Age, Median, Mean (SD)	78.5, 76.04 ± 12.81
Urban or Metropolitan	41455 (75.72%)
**Comorbidities**	
Myocardial Infarction	15607 (28.51%)
Peripheral Vascular Disease	6527 (11.92%)
Cerebrovascular Disease	5327 (9.73%)
Hypertension	36338 (66.38%)
Dementia	6981 (12.75%)
Chronic Pulmonary Disease	23819 (43.51%)
Diabetes Mellitus	21020 (38.40%)
Renal Disease	14901 (27.22%)
Liver Disease	2224 (4.06%)
Cancer	54728 (14.04%)
**Outcomes**	
Death in hospital at index$	7512
30-day HF ED visits#	8415 (8.2%)
30-day HF rehospitalizations#	4899 (4.8%)
30-day death#	5839 (5.7%)
30-day HF ED visits/HF rehospitalizations/death#	19153 (18.8%)
1-year episodes used in the studyǁ	93552 (100%)
1-year HF ED visitsǁ	13835 (14.8%)
1-year HF rehospitalizationsǁ	8850 (9.5%)
1-year deathǁ	27225 (29.1%)
1-year HF ED visits/HF rehospitalizations/deathǁ	49910 (53.4%)

ED-emergency department, HF-heart failure, SD-standard deviation

$ Index episodes which ended with death in the hospital were excluded from the study

# Percentages expressed in terms of total 102063 episodes used for 30-day outcome

ǁ Percentages expressed in terms of total 93552 episodes used for 365-day outcome

### Model performance and validation

Across all performance measures and study endpoints, CatBoost demonstrated superior performance to logistic regression modelling, with all t-test p-values for AUC-ROC and AUC-PRC <0.001. Table 2 presents different measures for cross-validation performance for both the CatBoost and logistic regression models for comparison, presented for the primary study endpoints. At 30-day and 1-year follow-up in the cross-validation, the AUC-ROC for the primary endpoint of HF ED visit, HF hospital readmission or death was superior for CatBoost over logistic regression model. The AUC-PRC and precision values for 30-day and 1-year endpoint were similarly higher for CatBoost model. Corresponding AUC-ROC values for the endpoint of all-cause death alone at 30-days and 1-year follow-up, along with respective AUC-PRC and precision values were higher for CatBoost model as well. Table 3 presents this same data for different measures of holdout validation performance comparing the two modelling approaches. All values demonstrated superiority of CatBoost over logistic regression modelling, with DeLong test p-values <0.01 for all comparisons.

**Table 2:** 5-fold cross-validation performance and statistical comparison between CatBoost and Logistic regression models.

Outcome	Metrics	CatBoost	Logistic Regression	t-statistic	p-value
**30-day**	N (Training set)	65320	65320		
	N (Test set)	16330	16330		
HF ED visit/HF rehospitalization or death (outcome rate: 18.8%)	AUC-ROC, mean (SD)	73.11 (0.19)	64.12 (0.18)	-64.58	<0.01
	AUC-PRC, mean (SD)	46.64 (0.71)	32.52 (0.51)	-29.46	<0.01
	Accuracy, mean (SD)	80.72 (0.42)	67.58 (0.14)	-75.69	<0.01
	Precision, mean (SD)	48.15 (1.45)	28.91 (0.18)	-26.81	<0.01
	Recall, mean (SD)	34.07 (1.33)	49.87 (0.8)	17.17	<0.01
	Specificity, mean (SD)	91.5 (0.75)	71.67 (0.31)	-56.19	<0.01
Death (outcome rate: 5.7%)	AUC-ROC, mean (SD)	82.29 (0.51)	71.17 (0.24)	-54.70	<0.01
	AUC-PRC, mean (SD)	45.04 (2.5)	19.81 (0.57)	-18.12	<0.01
	Accuracy, mean (SD)	88.03 (1.04)	75.94 (0.82)	-13.39	<0.01
	Precision, mean (SD)	25.54 (1.98)	12.59 (0.31)	-11.66	<0.01
	Recall, mean (SD)	56.2 (0.67)	53.91 (1.32)	-2.52	0.07
	Specificity, mean (SD)	89.97 (1.15)	77.27 (0.93)	-12.65	<0.01
**1-year**	N (Training set)	59873	59873		
	N (Test set)	14968	14968		
HF ED visit/HF rehospitalization or death (outcome rate: 53.4%)	AUC-ROC, mean (SD)	75.49 (0.53)	68.01 (0.44)	-16.69	<0.01
	AUC-PRC, mean (SD)	78.21 (0.33)	70.45 (0.53)	-27.86	<0.01
	Accuracy, mean (SD)	66.99 (0.28)	62.53 (0.27)	-23.43	<0.01
	Precision, mean (SD)	75.35 (0.78)	69.24 (0.34)	-11.72	<0.01
	Recall, mean (SD)	56.7 (0.86)	53.57 (0.37)	-9.15	<0.01
	Specificity, mean (SD)	84.14 (0.3)	69.36 (0.5)	-39.53	<0.01
Death (outcome rate: 29.1%)	AUC-ROC, mean (SD)	73.87 (0.42)	49.92 (0.65)	-46.89	<0.01
	AUC-PRC, mean (SD)	77.46 (0.36)	64.68 (0.37)	-41.86	<0.01
	Accuracy, mean (SD)	59.5 (0.58)	42.58 (0.43)	-38.43	<0.01
	Precision, mean (SD)	70.66 (0.46)	61.35 (0.74)	-27.11	<0.01
	Recall, mean (SD)	80.26 (0.44)	66.04 (0.4)	-38.58	<0.01
	Specificity, mean (SD)	89.97 (1.15)	77.27 (0.93)	-12.65	<0.01

**Fig 3:**
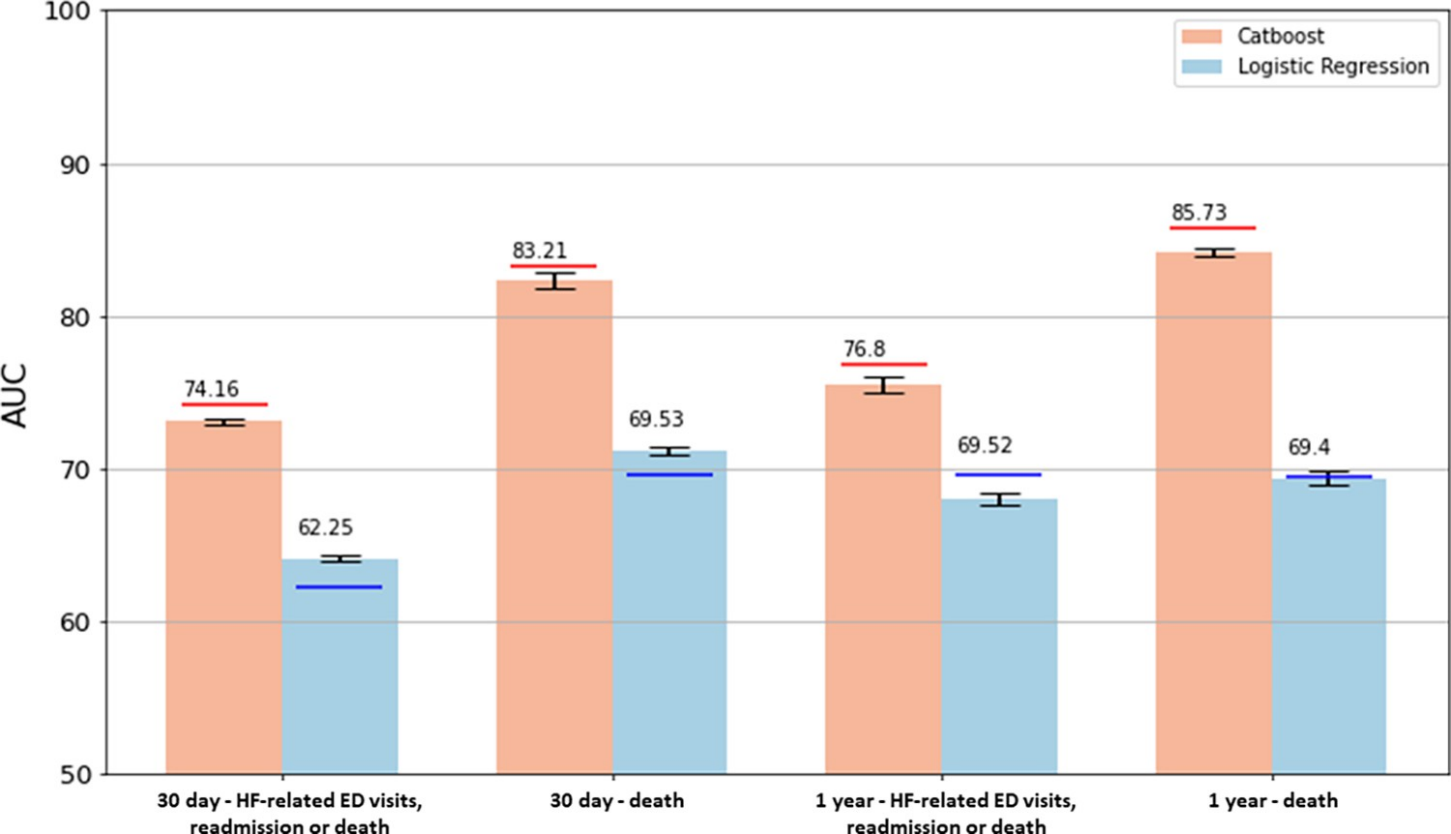
Comparison of area under receiver operating characteristic curve (AUC) performances between CatBoost and logistic regression models in cross-validation and holdout validation for the primary outcome of heart failure (HF)-related emergency department (ED) visits, HF hospital readmission or death and all-cause death alone (death) at 30-days and 1-year (365 days). Note that the Concordance index value of 0.5 indicates that the model performs no better than random chance and 1.0 implies perfect discrimination.

**Fig 4:**
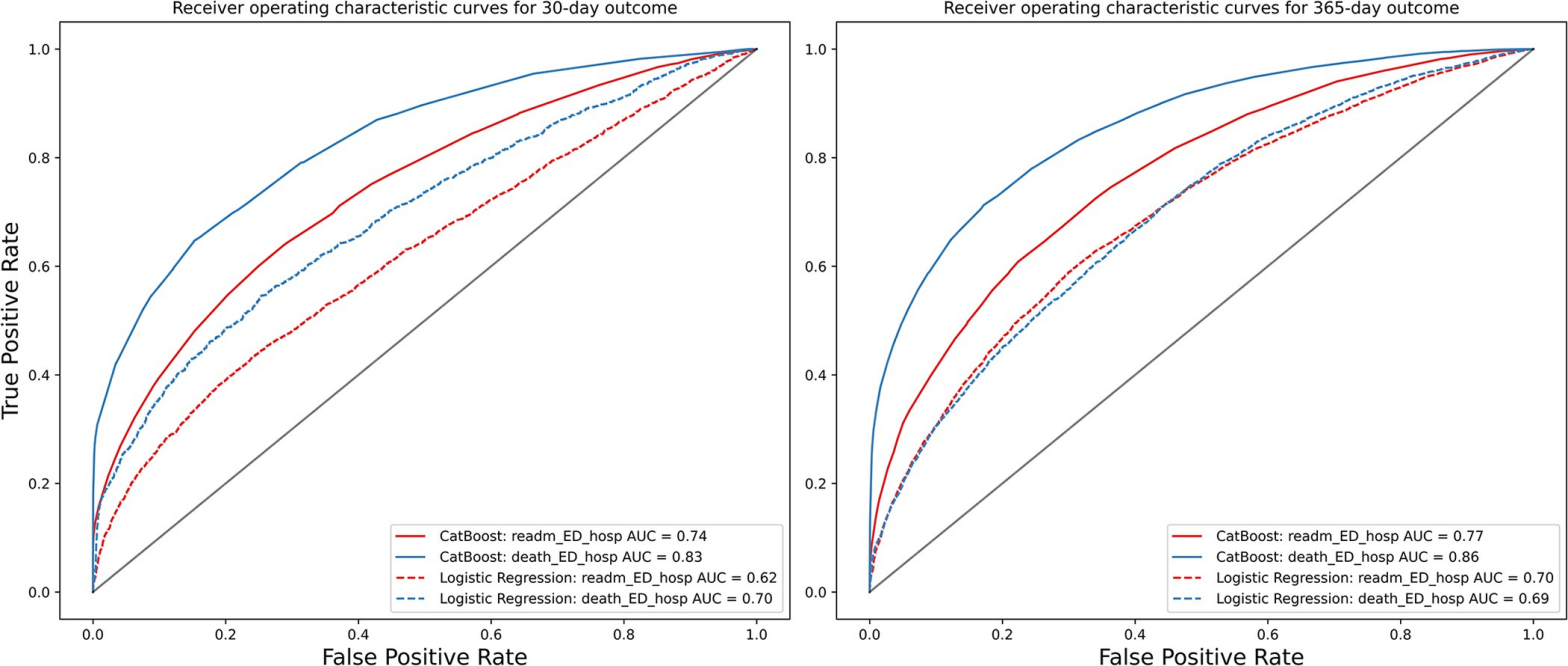
Receiver operating characteristic curves for 30-day (left) and 1-year (right) outcomes in holdout validation comparing machine learning (CatBoost) with logistic regression modelling for the primary outcome of heart failure (HF)-related emergency department (ED) visits, hospital readmission or death (death_readm_ED_hosp) and for HF-related ED visits and hospital readmission alone (readm_ED_hosp).

Model performance for CatBoost and logistic regression models were assessed for the secondary endpoint of all-cause death alone at 30-days and 1-year follow-up and presented in Table 3, again demonstrating superiority of CatBoost across all performance measures (with all p-values for AUC-ROC and AUC-PRC <0.001 for cross-validation and DeLong test <0.001 for holdout validation). Fig 3 shows a comparison of AUC-ROC performance values between CatBoost and logistic regression models in the cross-validation and holdout validation for the different study endpoints. Fig 4 presents corresponding ROC curves for 30-day and 1-year outcomes in holdout validation, again demonstrating superior performance of CatBoost modeling. Calibration plots describing agreement between predicted probabilities and observed proportions for different study endpoints are presented in S5 Fig. Feature importance for the primary study outcomes is presented in Table 4, and for all study outcomes in S6 Fig. CatBoost demonstrated superior performance to deep neural network, random forest and support vector machine modeling (S2 Table). S3 Table demonstrates performance of models trained on healthcare encounters ranging from fiscal years 2004 to 2012 on temporally disjoint holdout set ranging from 2013 to 2016, with superior performance of CatBoost compared using DeLong Test p-value <0.001 for all time-points and outcomes. Compared to the random data split evaluation, the CatBoost model with temporal data split exhibited lower AUC-ROC scores across various time periods and outcomes, with reductions for the primary endpoint of HF ED visit, HF hospital readmission or death of 7.86% at 30-days and 4.27% at 1-year follow-up.

**Table 3:** Holdout evaluation of performance on holdout test set between CatBoost and Logistic regression models. *DeLong Test p-values <0.001 for all time-points and outcomes.

Outcome	Metrics	30-day		1-year	
HF ED visit/HF rehospitalization or death (outcome rate: 30-day: 18.8%; 1-year: 53.4%)		CatBoost	Logistic Regression	CatBoost	Logistic Regression
	N (Training set)	81650	81650	74841	74841
	N (Test set)	20413	20413	18711	18711
	AUC-ROC*	74.16 [73.18–75.11]	62.25 [61.25–63.18]	76.8 [76.1–77.47]	69.52 [68.77–70.26]
	AUC-PRC	48.71 [47.03–50.47]	33.27 [31.81–34.6]	79.51 [78.73–80.25]	71.17 [70.23–72.05]
	Accuracy	81.95 [81.41–82.49]	66.98 [66.36–67.62]	67.01 [66.35–67.71]	63.25 [62.56–63.94]
	Precision	53.05 [50.9–55.32]	27.6 [26.66–28.58]	78.6 [77.57–79.67]	70.32 [69.4–71.28]
	Recall	33.39 [31.92–34.79]	46.8 [45.21–48.37]	52.44 [51.48–53.49]	53.84 [52.86–54.86]
	Specificity	93.17 [92.84–93.49]	71.64 [70.98–72.34]	83.68 [83.01–84.39]	74.02 [73.08–75.02]
**Death** (outcome rate: 30-day: 5.7%; 1-year: 29.1%)					
	AUC-ROC*	83.21 [81.83–84.41]	69.53 [67.98–71.18]	85.73 [85.14–86.29]	69.4 [68.57–70.26]
	AUC-PRC	45.94 [42.89–48.79]	19.69 [17.44–21.87]	76.61 [75.62–77.6]	50.24 [48.92–51.7]
	Accuracy	86.87 [86.43–87.29]	76.11 [75.53–76.68]	79.48 [78.93–80.05]	64.51 [63.85–65.24]
	Precision	23.75 [22.08–25.41]	12.01 [11.18–12.86]	63.26 [62.05–64.48]	42.28 [41.22–43.36]
	Recall	58.56 [55.88–61.18]	50.17 [47.42–52.9]	70.34 [69.08–71.6]	60.09 [58.88–61.36]
	Specificity	88.59 [88.2–88.99]	77.68 [77.09–78.26]	83.24 [82.6–83.87]	66.33 [65.55–67.16]

**Table 4:** Top features associated with the primary endpoints of 30-day and 1-year heart failure (HF) Emergency Department (ED) visit, HF rehospitalization or death using CatBoost modeling.

30-day	1-year
Discharge status/location of most recent inpatient visit	Discharge status/location of most recent inpatient visit
Primary and secondary diagnosis recorded in last physician office visit	Age
Trend of a primary and secondary diagnosis in ED visits over time	Trend of a primary and secondary diagnoses in ED visits over time
Most responsible diagnosis of most recent ED visit	Number of HF ED visits during observation window
Age	Postal Address
Most responsible diagnosis of most recent hospitalization	Most responsible diagnosis of most recent hospitalization
Postal Address	Primary and secondary diagnoses recorded in last physician office visits
Encounter for palliative care provider	Most responsible diagnosis of most recent ED visit

The results of our secondary analysis, examining prediction models for HF hospital readmission or all-cause death among patients hospitalized for HF (excluding HF ED visits), are presented in S4 Table and S5 Table, respectively. Consistent with the primary analysis that incorporated HF ED visit data, the AUC-ROC performance for CatBoost was consistently superior to that of logistic regression in this subgroup. CatBoost model performance metrics comparing subgroups of patients with different underlying HF etiologies and concomitant diagnoses are presented in S6 Table and S7 Fig.

## Discussion

The main findings of this study are 1) an ML-based tool utilizing a feature extraction method based on ‘deep feature synthesis’ can provide superior precision compared with logistic regression modelling for the prediction of HF ED visits, readmission to hospital or death for patients visiting the ED or hospitalized with HF from administrative health data on a large (population) scale, 2) this model was superior to logistic regression (and other advanced modelling techniques) for predicting HF outcomes at both 30-days and 1-year after discharge, leveraging multiple data sources from administrative health databases across a large regional healthcare network, and 3) ML-based modeling was feasible and demonstrated good performance with the inclusion of HF ED visits as an index event and important follow-up outcome. Taken together, these findings illustrate the value of ML-based modelling for improving adverse outcome prediction in patients hospitalized for HF from administrative health records data, and further illustrates the value of HF ED visits when assessing risk among HF patients. This approach may serve as a model that can be implemented and used with electronic medical record systems across other regional care providers to facilitate efficient and appropriate risk stratification of patients hospitalized with HF using administrative health data on a broader population level for HF patients, who have historically presented risk stratification challenges despite having a high rate of adverse outcomes and being a source of significant healthcare resource utilization.

While earlier studies have shown promise, subsequent reports examining ML-based approaches have demonstrated more limited performance improvement over conventional statistical methods for predicting the risk of HF readmission in patients discharged from hospital [20–22]. A study from Mortazavi, et al., assessed the predictive utility of machine learning techniques for the prediction of 30-day HF readmission among patients enrolled in the Telemonitoring to Improve Heart Failure Outcomes (Tele-HF) clinical trial [11]. This study demonstrated improved prediction of ML-based approaches compared with logistic regression modeling, with a random survival forest model performing best (C-statistic 0.678 versus 0.543, respectively). However, this study was limited by a small sample of patients with HF (N = 977) participating in a clinical trial environment, making its applicability to a large community HF population uncertain. Frizzell, et al., evaluated multiple ML algorithms to predict the risk of readmission to hospital within 30-days of HF hospitalization using data from the ‘Get With the Heart Failure Guidelines’ registry linked with Medicare data, and found no improvement compared with logistic regression modelling [6]. Desai, et al., evaluated multiple ML approaches to predict 1-year adverse HF outcomes including all-cause mortality and HF hospitalization (although not hospital readmission), for 9,502 Medicare enrolled patients with HF from 2 healthcare provider networks [9]. They also reported limited predictive efficacy compared with logistic regression modeling, however noted that the addition of select electronic medical record data to insurance-claims based data enhanced model performance. Notably, Christodoulou, et al., conducted a meta-analysis that included 71 studies that evaluated the utility of ML-based approaches for clinical risk prediction modeling across different diseases and found that there was no improved discrimination compared with conventional statistical modeling techniques [23]. It is noteworthy that success of such prediction models depends not only on the choice of learning algorithms, but also on structure of data sources and the methods of feature extraction.

Newer studies using more modern techniques such as deep learning have demonstrated more encouraging results using ML-based models to readmission following hospital discharge for HF patients. Golas, et al., used deep unified networks modeling to predict 30-day all-cause readmission following HF hospitalization using structured and unstructured electronic medical record data from a large urban healthcare networ [24]. Their model reported an AUC of 0.705, representing an improvement compared with previous studies examining AI-based approaches and superior to logistic regression. Sarijaloo, et al., recently reported an AUC of 0.76 using ML-based approach to predict a longer follow-up period of 90-day risk of HF readmission from electronic healthcare records in a smaller cohort of 3,189 patients from a single center [22]. Bat-Erdene et al. used a deep learning-based risk prediction model for HF rehospitalization during 6, 12 and 24-month follow that included 13,104 patients from the Korean Acute Myocardial Infarction-National Institutes of Health registry [17]. The proposed model outperformed logistic regression, support vector machine, AdaBoost, gradient boosting machine, and random forest and had a high level of accuracy, with an area under the curve, precision, recall, specificity, and F1 score of 99.37%, 99.90%, 96.86%, 98.61%, 99.49%, and 97.73%, respectively. Their model used a more select HF population of patients hospitalized for acute myocardial infarction, whereas our larger cohort included patients with all HF etiologies. Further, their model did not demonstrate improved performance compared with CatBoost when applied to our study’s dataset. Our study adds to this growing body of literature demonstrating superior performance of more modern ML-based approaches by including one of the largest (>50,000) unselected populations of HF patients visiting the ED or admitted to hospital for HF looking at both short-term (30-day) and longer-term (1-year) outcomes using administrative health data across a large geographic territory serviced by a single healthcare authority. Rahman et al. evaluated ED readmissions of discharged HF patients using ML-modelling using electronic health record data. Three feature selection techniques were studied along with 13 classical ML models using five-fold cross-validation [25]. The stacking ML model provided an accuracy, precision, recall, specificity, F1-score, and area under the curve (AUC) of 89.41%, 90.10%, 89.41%, 87.83%, 89.28%, and 0.881, respectively. HF ED visits are increasingly recognized as an important marker of worsening HF [26,27]

Earlier studies have identified input features (predictors) for their risk prediction model of HF readmissions based on expert domain knowledge or review of existing literature. Typically, these included demographic characteristics, HF-specific variables (such as left ventricular function) and pre-established composite scores [9,10]. In contrast, in our study, we automatically generated features using all the relevant clinical entries that were historically recorded in the administrative databases. This approach generated thousands of novel features based on aggregation and transformations of multiple clinical records, that were not typically used in earlier studies and might have contributed to improved performance of our models. The most predictive feature in our analysis was the discharge status or destination of the previous HF hospitalization, for example to home versus another healthcare facility. This novel feature has not been described in prior literature and may demonstrate the incremental value of leveraging large administrative healthcare databases to predict future adverse events in this population. Further, we used a gradient boosting algorithm to learn the model, which is one of the most powerful algorithms in the field of ML [28]. In gradient boosting, an ensemble of weak learners is used to improve the performance of a ML model [29]. In particular, we used a depth-wise gradient boosting library CatBoost because it provided several desirable properties for challenges posed by our dataset and modelling, such as native support for categorical features, training on multiple GPUs and handling the missing values natively [14]. Our findings illustrate the value of domain agnostic, automated feature engineering and gradient boosted ML methods using administrative health records data for improving adverse outcome prediction of patients hospitalized for HF. Also of interest is that the risk prediction algorithm for our study had better performance for 1-year than 3-day outcomes. This may be due to slightly different samples (more patients had incomplete follow-up for the 1-year horizon).

### Limitations

While our analysis includes a comprehensive dataset, there are some limitations that warrant mention. A number of inherent limitations exist regarding the use administrative healthcare data, including a lack of validation and missing values. Model performance hinges on various factors such as the availability of information rich features, the size of the training dataset, and not just the selection of learning algorithms. Administrative healthcare data differ from electronic medical records as they primarily serve billing purposes for hospitalizations, emergency department visits, and outpatient encounters, lacking detailed clinical information like physical measurements, laboratory tests, vital signs and imaging data. Multiple categories of HF-related data variables, including electrocardiography, cardiovascular imaging, cardiac device, and invasive hemodynamic and angiographic data were not available for inclusion for this analysis. Complete laboratory and pharmacy data were also not available for inclusion in our analysis. Patient race and ethnicity are recognized as important determinants of HF outcomes, however racial and ethnicity data was unfortunately also not available from this administrative healthcare dataset. Our study included a broad range of patients with HF admitted to hospital and did not assess subpopulations such as patients with HF with preserved, mildly reduced, or reduced ejection fraction. Furthermore, the outcomes of cardiac transplantation and left ventricular assist device placement were not included in this analysis because of their relative rarity. Our analysis used an observation window of 1-year lookback prior to the discharge date of the index event, and a different window duration may have influenced the findings. Our study reveals that CatBoost predictive modeling, relying solely on administrative records, exhibits good predictive performance in terms of AUROC. Before model implementation can occur, it is important to explore additional domain-specific evaluation metrics that account for factors such as resource utilization and the relative costs associated with false positive and false negative classifications [30]. These considerations will aid assessing the model’s suitability for real-world application in healthcare settings.

## Conclusion

Using an ML-based approach that applied a deep feature synthesis method for automated feature engineering for pooling of patient information from multiple data sources, we developed and validated an algorithm predicting 30-day and 1-year risk of HF ED visits, HF rehospitalization and all-cause mortality among patients visiting the ED or hospitalized for HF. This model used administrative healthcare records from a large region serviced by a common healthcare authority and demonstrated superior prognostic utility and precision compared with more conventional logistic regression modelling. Further research is needed to determine if this approach could be implemented across large regional healthcare systems to reduce adverse events post discharge for HF decompensation using administrative health data on a large scale (population) level.

## Supporting information

S1 Table Study variables and grouping of variables included in the analysis. (DOCX)

S2 Table 5-fold cross-validation performance and statistical comparison between CatBoost and logistic regression models for heart failure (HF) rehospitalization or death among patients hospitalized with HF. (DOCX)

S3 Table Holdout validation evaluation of performance on test set between CatBoost and Logistic regression models for heart failure (HF) rehospitalization or death among patients hospitalized with HF. *DeLong Test p-value <0.001 for both time-points.
(DOCX)

S4 Table Holdout evaluation of performance on test set using neural network, random forest and support vector machine (SVM) models. (DOCX)

S5 Table Evaluation of performance of models trained on healthcare encounters ranging from fiscal years 2004 to 2012 on temporally disjoint holdout set ranging from 2013 to 2016. *DeLong Test p-value <0.001 for all time-points and outcomes.
(DOCX)

S6 Table Holdout evaluation of performance for all time-points and outcomes for subgroups of patients with specific diagnoses for heart failure etiologies and concomitant conditions in the holdout test set. These diagnoses were identified using the ICD-10 codes in the diagnosis fields of healthcare episode in the administrative records.
(DOCX)

S1 Fig Episode definition algorithm used to group continuous healthcare encounters. (INP: Inpatient hospitalizations; ED: Emergency department visit).
(DOCX)

S2 Fig Flowchart describing samples sizes (index events) in the secondary analysis cohort (for the outcomes of 30-day and 1-year heart failure (HF) hospital readmission or death), used for training and for validating the models for each outcome, after applying the episode creation and study exclusion criteria. (DOCX)

S3 Fig Feature extraction from the relational database structure of administrative health data. (DOCX)

S4 Fig Study outcome distributions by year. (DOCX)

S5 Fig Bar plots showing the calibration of CatBoost Models. Green to Red indicates low- to high-risk groups based on 20% quantile steps of predicted probability score. Gray bar suggests unreliable estimate due to <10 encounters in the risk group. ED-emergency department, HF-heart failure.
(DOCX)

S6 Fig Feature importance and SHAP (SHapley Additive exPlanations) analyses figures for the models presented in the paper. The feature names are presented in the format “mathematical_operation(database.variable)”. For example, LAST (Hospitalizations_visits.Discharge status) refers to the discharge status assigned during a patient’s last inpatient visit. Please refer to S1 Table for the details, grouping and source database of study variables included in the analysis.
(DOCX)

S7 Fig Bar plot representing the holdout performance in terms of AUROC scores for patient subgroups with specific heart failure etiologies and concomitant conditions. (DOCX)
