# Supplementary material for: Machine Learning For Risk Prediction After Heart Failure Emergency Department Visit or Hospital Admission Using Administrative Health Data
Source: PLOS Digit Health. 2024 Oct 25;3(10):e0000636. doi: 10.1371/journal.pdig.0000636 (PMC11508085; doi:10.1371/journal.pdig.0000636)
Supplement: S1 Table — (DOCX) [file pdig.0000636.s001.docx]

**Supplementary Table 1.** Study variables and grouping of variables included in the analysis.

| **inp: DAD Database (Hospitalizations/Inpatient)** | |
| --- | --- |
| **Variable Name** | **Description** |
| ***obs_id, episode_order*** | ***Count order of HF episodes for a patient*** |
| IAB_Rcpt_Age_SD | Patient age during the encounter |
| HLTH_DX_CODE_MR | Main diagnosis |
| HLTH_DX_CODE_MR_OTH (25) | Secondary diagnoses (up to 25) |
| HLTH_DX_TYPE_CODE_MR_OTH (25) | whether condition was present on admission or occurred after admission (up to 25) |
| FAC_INST_ID_TRNSFR_FROM_MC | Facility Institution Identifier Transfer From |
| FAC_INST_ID_TRNSFR_TO_MC | Facility Institution Identifier Transfer To |
| HLTH_STATUS_AUTOL_FLAG | Whether the patient was transfused with his/her own blood |
| HLTH_STATUS_BLD_OTH_CMPTS_FLAG | Components: whether the patient received via transfusion |
| HLTH_STATUS_BLD_RED_CELLS_FLAG | Red Cells: whether the patient received via transfusion |
| HLTH_STATUS_BLOOD_ALBUMIN_FLAG | Albumin: whether the patient received via transfusion |
| HLTH_STATUS_BLOOD_PLASMA_FLAG | Plasma: whether the patient received via transfusion |
| HLTH_STATUS_BLOOD_PLAT_FLAG | Platelets: whether the patient received via transfusion |
| HLTH_STATUS_BLOOD_TRNSFSN_FLAG | Transfusion: whether the patient received via transfusion |
| Prvd_Spec_Grp_MR | Most responsible provider speciality |
| Prvd_Spec_Grp_MR_Int (20) | Provider specialities for interventions provided (up to 20) |
| SE_ADMIT_VIA_AMBLC_FLAG | Arrival by ambulance flag |
| SE_AMBLC_TYPE_CODE | Whether patient arrives via ambulance to the health care facility |
| SE_INTRV_CODE_PR | Primary Intervention code |
| SE_INTRV_CODE_PR_OTH (20) | Interventions provided (up to 20) |
| SEPI_ALT_LVLOC_LOS_DAYS | The number of days spent in an acute care bed while awaiting placement |
| SEPI_MAIN_SRVC_CODE | Focus of the treatment provided during a hospital stay. |
| Sepi_Scu_Dura_Days_1 | Length of stay in days, in a special care unit. |
| SEPI_SPEC_CARE_ADMIT_UNIT_CODE1 | Primary type of special care unit where the patient receives critical care |
| SEPI_SPEC_CARE_ADMIT_UNIT_CODE (3) | Secondary special care units (up to 3) |
| Prvd_Role_Code (5) | Role of the health care providers (up to 5) |
| *stay_duration* | *Number of days in hospital* |
| *episode_duration* | *Number of days during current episode(includes transfers)* |
| SEPI_DISCH_DISPOS_CODE | Discharge status |
| REC_SUBM_FAC_ID | Hospital ID |
| SE_CODE_VER_NUM_CODE | Intervention coding scheme |
| SEPI_ADMIT_CAT_CODE | Admission Category Code |
| SEPI_CMG_CODE | Case Mix Group Code |
| SEPI_ELOS_CPLX_DAYS | Expected Length of Stay Complexity Days (derived by Alberta or CIHI) |
| SEPI_RIW_VALUE | Resource Intensity Weight Value. |
| Total_Cost | Total Cost provided by Alberta Health Services. |
| SEPI_CPLX_LVL_CODE_CIHI | CIHI Code? |
| SEPI_START_DATE | Service Start Date |
| SE_INTRV_DATE_PR | Primary Intervention date |
| SEPI_END_DATE | Service End Date |
| *6 mon, 3 mon, 1 mon and 'during episode' indicators* | *Whether encounter happened during 6, 3 or 1 months prior to index episode or during the index episode* |
|  |  |
| **amb: NACRS Database (Emergency/Ambulatory)** | |
| **Variable Name** | **Description** |
| ***obs_id, episode_order*** | ***Count order of HF episodes for a patient*** |
| IAB_Rcpt_Age_SD | Patient age during the encounter |
| HLTH_DX_CODE_MR | Main diagnosis |
| HLTH_DX_CODE_MR_OTH (10) | Secondary diagnoses (up to 10) |
| Prvd_Spec_Grp | Most responsible provider speciality |
| SE_AMBLC_TYPE_CODE | Whether patient arrives via ambulance to the health care facility |
| SEPI_MIS_PRIM_CODE | Whether the encounter is emergency |
| SE_INTRV_CODE_PR | Primary Intervention code |
| SE_INTRV_CODE_PR_OTH (10) | Interventions provided (up to 10) |
| SEPI_DISPOS_CODE | Discharge status |
| REC_SUBM_FAC_ID | Facility ID |
| SEPI_CACS_CODE | grouper to create homogeneous patient clusters |
| SEPI_CACS_RIW_VALUE | Resource Intensity Weight Value. |
| TOTAL_COST | Total Cost provided by Alberta Health Services. |
| SEPI_ACCS_GRP_CODE | ACCS Code? |
| SEPI_START_DATE | Service Start Date |
| *6 mon, 3 mon, 1 mon and 'during episode' indicators* | *Whether encounter happened during 6, 3 or 1 months prior to index episode or during the index episode* |
|  |  |
| **clm : CLAIMS Database (Physician Billing)** | |
| **Variable Name** | **Description** |
| HLTH_DX_ICD9X_CODE_1 | Main diagnosis |
| HLTH_DX_ICD9X_CODE_123 (3) | Secondary diagnoses (up to 3) |
| DELV_SITE_FUNCTR_CODE_CLS | Identifies ICU, Surgical, Clinic, Practitioner's Office |
| HLTH_SRVC_CCPX_CODE | Primary Intervention code |
| Prvd_Spec_Grp | Most responsible provider speciality |
| PGM_APP_IND | Alternate Payment Plan Indicator |
| FRE_ACTUAL_PAID_AMT | Total amount paid for the claim |
| CAE_SYS_AMT | Assessment amount determined by the system |
| SE_END_DATE | Service end date |
| *6 mon, 3 mon and 1 mon indicators* | *Whether encounter happened during 6, 3 or 1 months prior to index episode.* |
|  |  |
| **AHCIP registry** |  |
| **Variable Name** | **Description** |
| ADDR_POSTAL_CODE_FYE | Address Postal Code |
| PERS_GENDER_CODE | Biological sex |
| PERS_SOCIO_ECON_STATUS_FYE | Socio Economic Status |
| Rcpt_Birth_Year | Year of Birth |
| mhi2010 | Household income |
| urban | Urbanicity |
| Eth_Grp | Ethnicity Group |
| Eth_Grp_HP | Ethnicity Group - Highly Predictive |
| obs_year | Fiscal Year |
|  |  |
| **Legend** | |
| Original Variables | VAR |
| Derived Variables | *Var* |
| Number of Variables | VAR (num) |
|  |  |
| **Feature Type** | Color coding |
| Diagnoses |  |
| Demographics |  |
| Intervention |  |
| Discharge |  |
| Admin |  |
| Date related |  |
