# Supplementary material for: Machine Learning For Risk Prediction After Heart Failure Emergency Department Visit or Hospital Admission Using Administrative Health Data
Source: PLOS Digit Health. 2024 Oct 25;3(10):e0000636. doi: 10.1371/journal.pdig.0000636 (PMC11508085; doi:10.1371/journal.pdig.0000636)
Supplement: S2 Table — (DOCX) [file pdig.0000636.s002.docx]

**Supplementary Table 2.** 5-fold cross-validation performance and statistical comparison between CatBoost and logistic regression models for heart failure (HF) rehospitalization or death among patients hospitalized with HF.

| **Outcome** | **Metrics** | **CatBoost** | **Logistic Regression** | **t-statistic** | **p-value** |
| --- | --- | --- | --- | --- | --- |
| 30-day | N (Training set) | 34958 | 34958 |  |  |
|  | N (Test set) | 8740 | 8740 |  |  |
| HF rehospitalization or death | AUC-ROC, mean (SD) | 69.03 (1.04) | 65.11 (1.18) | -5.26 | <0.01 |
|  | AUC-PRC, mean (SD) | 31.44 (1.94) | 25.56 (1.13) | -4.46 | 0.01 |
|  | Accuracy, mean (SD) | 74.49 (0.77) | 69.01 (0.8) | -42.50 | <0.01 |
|  | Precision, mean (SD) | 26.43 (0.59) | 22.65 (0.6) | -9.11 | <0.01 |
|  | Recall, mean (SD) | 46.01 (2.43) | 50.16 (1.61) | 2.86 | 0.05 |
|  | Specificity, mean (SD) | 79.12 (1.21) | 72.08 (1.04) | -38.33 | <0.01 |
| 1-year | N (Training set) | 31860 | 31860 |  |  |
|  | N (Test set) | 7965 | 7965 |  |  |
| HF rehospitalization or death | AUC-ROC, mean (SD) | 71.81 (0.18) | 67.35 (0.48) | -18.62 | <0.01 |
|  | AUC-PRC, mean (SD) | 70.65 (0.3) | 65.83 (0.37) | -25.16 | <0.01 |
|  | Accuracy, mean (SD) | 65.69 (0.34) | 62.74 (0.27) | -10.77 | <0.01 |
|  | Precision, mean (SD) | 66.92 (0.61) | 63.25 (0.39) | -8.16 | <0.01 |
|  | Recall, mean (SD) | 57.83 (1.09) | 55.19 (0.58) | -7.26 | <0.01 |
|  | Specificity, mean (SD) | 73.1 (1.11) | 69.84 (0.63) | -5.07 | <0.01 |
