# Supplementary material for: Machine Learning For Risk Prediction After Heart Failure Emergency Department Visit or Hospital Admission Using Administrative Health Data
Source: PLOS Digit Health. 2024 Oct 25;3(10):e0000636. doi: 10.1371/journal.pdig.0000636 (PMC11508085; doi:10.1371/journal.pdig.0000636)
Supplement: S3 Table — *DeLong Test p-value <0.001 for both time-points. (DOCX) [file pdig.0000636.s003.docx]

**Supplementary Table 3.** Holdout validation evaluation of performance on test set between CatBoost and Logistic regression models for heart failure (HF) rehospitalization or death among patients hospitalized with HF. *DeLong Test p-value <0.001 for both time-points.

| **Metrics** | **30-day** | | **1-year** | |
| --- | --- | --- | --- | --- |
| **HF rehospitalization or death** | **CatBoost** | **Logistic Regression** | **CatBoost** | **Logistic Regression** |
| N (Training set) | 43698 | 43698 | 39825 | 39825 |
| N (Test set) | 10925 | 10925 | 9957 | 9957 |
| AUC-ROC* | 69.99 [68.61-71.45] | 63.27 [61.85-64.72] | 73.63 [72.73-74.61] | 67.99 [67.03-68.99] |
| AUC-PRC | 31.39 [28.96-33.88] | 24.62 [22.83-26.63] | 72.45 [71.17-73.8] | 66.56 [65.19-68.07] |
| Accuracy | 77.82 [77.08-78.55] | 68.23 [67.42-69.04] | 66.79 [65.88-67.7] | 63.42 [62.48-64.37] |
| Precision | 29.71 [26.8-31.96] | 21.85 [20.45-23.24] | 70.04 [68.39-71.67] | 64.18 [62.82-65.63] |
| Recall | 42.65 [40.06-45.1] | 49.18 [46.58-51.69] | 54.99 [53.55-56.29] | 55.51 [54.19-56.89] |
| Specificity | 83.55 [82.87-84.21] | 71.33 [70.41-72.2] | 77.88 [76.92-78.85] | 70.86 [69.61-72.13] |
