# Supplementary material for: Machine Learning For Risk Prediction After Heart Failure Emergency Department Visit or Hospital Admission Using Administrative Health Data
Source: PLOS Digit Health. 2024 Oct 25;3(10):e0000636. doi: 10.1371/journal.pdig.0000636 (PMC11508085; doi:10.1371/journal.pdig.0000636)
Supplement: S4 Table — (DOCX) [file pdig.0000636.s004.docx]

**Supplementary Table 4.** Holdout evaluation of performance on test set using neural network, random forest and support vector machine (SVM) models.

| **Outcome** | **Metrics** | **30-day** | | | **1-year** | | |
| --- | --- | --- | --- | --- | --- | --- | --- |
| **HF ED visit/HF rehospitalization or death** |  | **Neural Network** | **Random Forest** | **SVM** | **Neural Network** | **Random Forest** | **SVM** |
|  | N (Training set) | 81650 | 81650 | 81650 | 74841 | 74841 | 74841 |
|  | N (Test set) | 20413 | 20413 | 20413 | 18711 | 18711 | 18711 |
|  | AUC-ROC | 59.33 | 60.58 | 56.99 | 68.43 | 66.27 | 68.86 |
|  | AUC-PRC | 40.6 | 28.28 | 41.88 | 74.14 | 74.6 | 75.29 |
|  | Accuracy | 63.39 | 79.07 | 81.55 | 62.8 | 62.25 | 63.9 |
|  | Precision | 24.7 | 30.48 | 61.25 | 66.59 | 63.2 | 65.75 |
|  | Recall | 46.44 | 9.01 | 4.62 | 60.77 | 70 | 67.5 |
|  | Specificity | 67.3 | 95.25 | 99.32 | 65.13 | 53.39 | 59.8 |
| **Death** |  |  |  |  |  |  |  |
|  | AUC-ROC | 71.74 | 67.31 | 62.88 | 68.21 | 65.1 | 67.27 |
|  | AUC-PRC | 35.36 | 26.99 | 41.56 | 58.32 | 49.23 | 52.62 |
|  | Accuracy | 75.81 | 94.01 | 94.61 | 60.87 | 69.42 | 73.11 |
|  | Precision | 12.78 | 39.7 | 66.5 | 39.81 | 46.37 | 63.47 |
|  | Recall | 55.39 | 9.08 | 11.56 | 67.35 | 32.42 | 17.87 |
|  | Specificity | 77.05 | 99.16 | 99.65 | 58.21 | 84.61 | 95.78 |
| **HF rehospitalization or death** |  |  |  |  |  |  |  |
|  | N (Training set) | 43698 | 43698 | 43698 | 39825 | 39825 | 39825 |
|  | N (Test set) | 10925 | 10925 | 10925 | 9957 | 9957 | 9957 |
|  | AUC-ROC | 64.27 | 61.68 | 57.9 | 68.77 | 65.11 | 68.95 |
|  | AUC-PRC | 41.51 | 23.1 | 31.94 | 71.5 | 69.66 | 69.9 |
|  | Accuracy | 63.05 | 85.51 | 85.95 | 63.36 | 60.92 | 63.14 |
|  | Precision | 20.42 | 30 | 47.62 | 61.96 | 59.45 | 67 |
|  | Recall | 56.5 | 2.55 | 2.61 | 63.22 | 60.94 | 47.2 |
|  | Specificity | 64.12 | 99.03 | 99.53 | 63.5 | 60.9 | 78.13 |
