# Supplementary material for: Machine Learning For Risk Prediction After Heart Failure Emergency Department Visit or Hospital Admission Using Administrative Health Data
Source: PLOS Digit Health. 2024 Oct 25;3(10):e0000636. doi: 10.1371/journal.pdig.0000636 (PMC11508085; doi:10.1371/journal.pdig.0000636)
Supplement: S5 Table — *DeLong Test p-value <0.001 for all time-points and outcomes. (DOCX) [file pdig.0000636.s005.docx]

**Supplementary Table 5.** Evaluation of performance of models trained on healthcare encounters ranging from fiscal years 2004 to 2012 on temporally disjoint holdout set ranging from 2013 to 2016. *DeLong Test p-value <0.001 for all time-points and outcomes.

| **Outcome** | **Metrics** | **30-day** | | **1-year** | |
| --- | --- | --- | --- | --- | --- |
| **HF ED visit/HF rehospitalization or death** |  | **CatBoost** | **Logistic Regression** | **CatBoost** | **Logistic Regression** |
|  | N (Training set) | 67415 | 67415 | 67415 | 67415 |
|  | N (Test set) | 34648 | 34648 | 26137 | 26137 |
|  | AUC-ROC* | 66.3 | 64.05 | 72.53 | 68.6 |
|  | AUC-PRC | 34.73 | 31.44 | 73.94 | 71.3 |
|  | Accuracy | 81.82 | 65.78 | 59.61 | 63.02 |
|  | Precision | 61.58 | 27.98 | 78.79 | 68 |
|  | Recall | 11.16 | 51.04 | 34.03 | 58.97 |
|  | Specificity | 98.37 | 69.23 | 89.35 | 67.73 |
| **Death** |  |  |  |  |  |
|  | AUC-ROC* | 79.54 | 72.19 | 75.34 | 69.82 |
|  | AUC-PRC | 37.59 | 23.86 | 59.16 | 52.34 |
|  | Accuracy | 87.56 | 71.03 | 70.93 | 63.91 |
|  | Precision | 25.73 | 12.22 | 51.73 | 43.36 |
|  | Recall | 52.99 | 59.16 | 60.7 | 62.43 |
|  | Specificity | 89.86 | 71.82 | 75.38 | 64.55 |
| **HF rehospitalization or death** |  |  |  |  |  |
|  | N (Training set) | 35097 | 35097 | 35097 | 35097 |
|  | N (Test set) | 19526 | 19526 | 14685 | 14685 |
|  | AUC-ROC* | 67.03 | 65.44 | 70.65 | 69.28 |
|  | AUC-PRC | 28.09 | 26.38 | 68.44 | 68.52 |
|  | Accuracy | 79.68 | 64.4 | 60.89 | 63.79 |
|  | Precision | 30.72 | 21.6 | 73.18 | 65.27 |
|  | Recall | 31.65 | 55.04 | 33.62 | 57.96 |
|  | Specificity | 87.85 | 66 | 87.83 | 69.55 |
