# Supplementary material for: Machine Learning For Risk Prediction After Heart Failure Emergency Department Visit or Hospital Admission Using Administrative Health Data
Source: PLOS Digit Health. 2024 Oct 25;3(10):e0000636. doi: 10.1371/journal.pdig.0000636 (PMC11508085; doi:10.1371/journal.pdig.0000636)
Supplement: S6 Table — These diagnoses were identified using the ICD-10 codes in the diagnosis fields of healthcare episode in the administrative records. (DOCX) [file pdig.0000636.s006.docx]

**Supplementary Table 6.** Holdout evaluation of performance for all time-points and outcomes for subgroups of patients with specific diagnoses for heart failure etiologies and concomitant conditions in the holdout test set. These diagnoses were identified using the ICD-10 codes in the diagnosis fields of healthcare episode in the administrative records.

| **30-day** | | | | | | | |
| --- | --- | --- | --- | --- | --- | --- | --- |
| **HF ED visit/HF rehospitalization or death** | | | | | | | |
| **Conditions** | **AUC-ROC** | **AUC-PRC** | **Accuracy** | **Precision** | **Recall** | **Specificity** | **N (test)** |
| Myocardial Infarction | 72.96 | 47.16 | 82.28 | 52.84 | 30.26 | 93.94 | 9076 |
| Peripheral Vascular Disease | 72.93 | 45.88 | 84.18 | 56.45 | 27.85 | 95.63 | 2232 |
| Cerebrovascular Disease | 72.45 | 43.12 | 85.02 | 52.11 | 25.61 | 95.75 | 1889 |
| Hypertension | 72.7 | 45.05 | 83.61 | 52.23 | 27.86 | 94.86 | 12506 |
| Dementia | 73.05 | 52.14 | 80.84 | 54.04 | 36.71 | 92.06 | 1707 |
| Chronic Pulmonary Disease | 73.96 | 48.93 | 82.12 | 52.05 | 31.84 | 93.41 | 8050 |
| Diabetes Mellitus | 73.21 | 45.83 | 82.21 | 49.77 | 30.57 | 93.34 | 7964 |
| Renal Disease | 74.21 | 52.86 | 79.31 | 51.44 | 37.18 | 90.6 | 4558 |
| Liver Disease | 74.88 | 52.47 | 82.3 | 54.93 | 30.71 | 94.19 | 678 |
| Cancer | 72.55 | 50.77 | 83.06 | 57.48 | 31.74 | 94.68 | 2491 |
| Atrial Fibrillation | 73.43 | 48.3 | 81.78 | 51.64 | 31.83 | 93.19 | 8242 |
| **Death** | | | | | | | |
| Myocardial Infarction | 82.28 | 47.81 | 86.6 | 23.99 | 59.02 | 88.32 | 9051 |
| Peripheral Vascular Disease | 84.89 | 56.23 | 84.72 | 26.8 | 67.92 | 85.99 | 2265 |
| Cerebrovascular Disease | 85.54 | 56.91 | 84.01 | 26.5 | 68.38 | 85.23 | 1883 |
| Hypertension | 83.48 | 47.79 | 86.77 | 23.68 | 61.08 | 88.3 | 12549 |
| Dementia | 74.26 | 45.75 | 63.85 | 19.14 | 71.28 | 62.93 | 1715 |
| Chronic Pulmonary Disease | 82.73 | 49.43 | 86.05 | 25.89 | 61.16 | 87.78 | 8171 |
| Diabetes Mellitus | 83.97 | 46.36 | 87.26 | 23.63 | 59.45 | 88.87 | 7929 |
| Renal Disease | 81.81 | 53.05 | 82.04 | 26.73 | 64.12 | 83.7 | 4466 |
| Liver Disease | 82.51 | 49.91 | 84.49 | 24.04 | 51.02 | 87.15 | 664 |
| Cancer | 81.61 | 53.73 | 80.27 | 27.52 | 65.95 | 81.77 | 2443 |
| Atrial Fibrillation | 83.12 | 50.36 | 85.77 | 24.31 | 61.94 | 87.33 | 8353 |

| **365-day** | | | | | | | |
| --- | --- | --- | --- | --- | --- | --- | --- |
| **HF ED visit/HF rehospitalization or death** | | | | | | | |
| **Conditions** | **AUC-ROC** | **AUC-PRC** | **Accuracy** | **Precision** | **Recall** | **Specificity** | **N (test)** |
| Myocardial Infarction | 75.03 | 78.19 | 65.86 | 76.8 | 51.6 | 82.18 | 8273 |
| Peripheral Vascular Disease | 74.31 | 78.09 | 64.05 | 78.22 | 46.81 | 84.53 | 2078 |
| Cerebrovascular Disease | 73.62 | 73.49 | 64.87 | 75.92 | 41.07 | 87.57 | 1651 |
| Hypertension | 74.7 | 76.53 | 65.59 | 76.53 | 47.36 | 84.74 | 11341 |
| Dementia | 69.72 | 78.63 | 61.97 | 76.13 | 53.5 | 74.72 | 1567 |
| Chronic Pulmonary Disease | 74.95 | 78.63 | 66.01 | 78.03 | 52.12 | 82.54 | 7525 |
| Diabetes Mellitus | 75.37 | 78.57 | 65.9 | 77.08 | 52.06 | 82 | 7234 |
| Renal Disease | 74.8 | 82.96 | 66.63 | 78.8 | 62.2 | 73.62 | 4112 |
| Liver Disease | 75.57 | 79.11 | 66.51 | 81.35 | 46.87 | 88.16 | 639 |
| Cancer | 73.95 | 79.55 | 63.16 | 79.32 | 47.19 | 83.97 | 2169 |
| Atrial Fibrillation | 75.34 | 79.16 | 66.38 | 77.64 | 53.83 | 81.42 | 7538 |
| **Death** | | | | | | | |
| Myocardial Infarction | 84.7 | 75.62 | 77.92 | 61.83 | 69.83 | 81.41 | 8369 |
| Peripheral Vascular Disease | 82.13 | 73.58 | 74.79 | 60.82 | 69.41 | 77.5 | 2031 |
| Cerebrovascular Disease | 82.37 | 70.78 | 74.15 | 56.41 | 70.53 | 75.76 | 1764 |
| Hypertension | 83.87 | 72.7 | 77.8 | 58.79 | 68.49 | 81.4 | 11391 |
| Dementia | 76.73 | 75.24 | 62.97 | 55.13 | 84.44 | 46.15 | 1507 |
| Chronic Pulmonary Disease | 84.17 | 75.44 | 76.78 | 60.36 | 71.06 | 79.32 | 7382 |
| Diabetes Mellitus | 85.11 | 75.32 | 79.45 | 63.48 | 67.88 | 84.15 | 7255 |
| Renal Disease | 83.59 | 80.03 | 72.52 | 61.67 | 78.43 | 68.73 | 4105 |
| Liver Disease | 80.94 | 75.51 | 75.64 | 62.13 | 69.52 | 78.71 | 628 |
| Cancer | 80.29 | 76.96 | 71.76 | 63.1 | 73.09 | 70.85 | 2227 |
| Atrial Fibrillation | 84.9 | 75.66 | 77.43 | 60.54 | 72.3 | 79.65 | 7479 |
