# Supplementary material for: Machine Learning For Risk Prediction After Heart Failure Emergency Department Visit or Hospital Admission Using Administrative Health Data
Source: PLOS Digit Health. 2024 Oct 25;3(10):e0000636. doi: 10.1371/journal.pdig.0000636 (PMC11508085; doi:10.1371/journal.pdig.0000636)
Supplement: S1 Fig — (INP: Inpatient hospitalizations; ED: Emergency department visit). (DOCX) [file pdig.0000636.s007.docx]

**Supplementary Figure 1**. Episode definition algorithm used to group continuous healthcare encounters. (INP: Inpatient hospitalizations; ED: Emergency department visit).

**
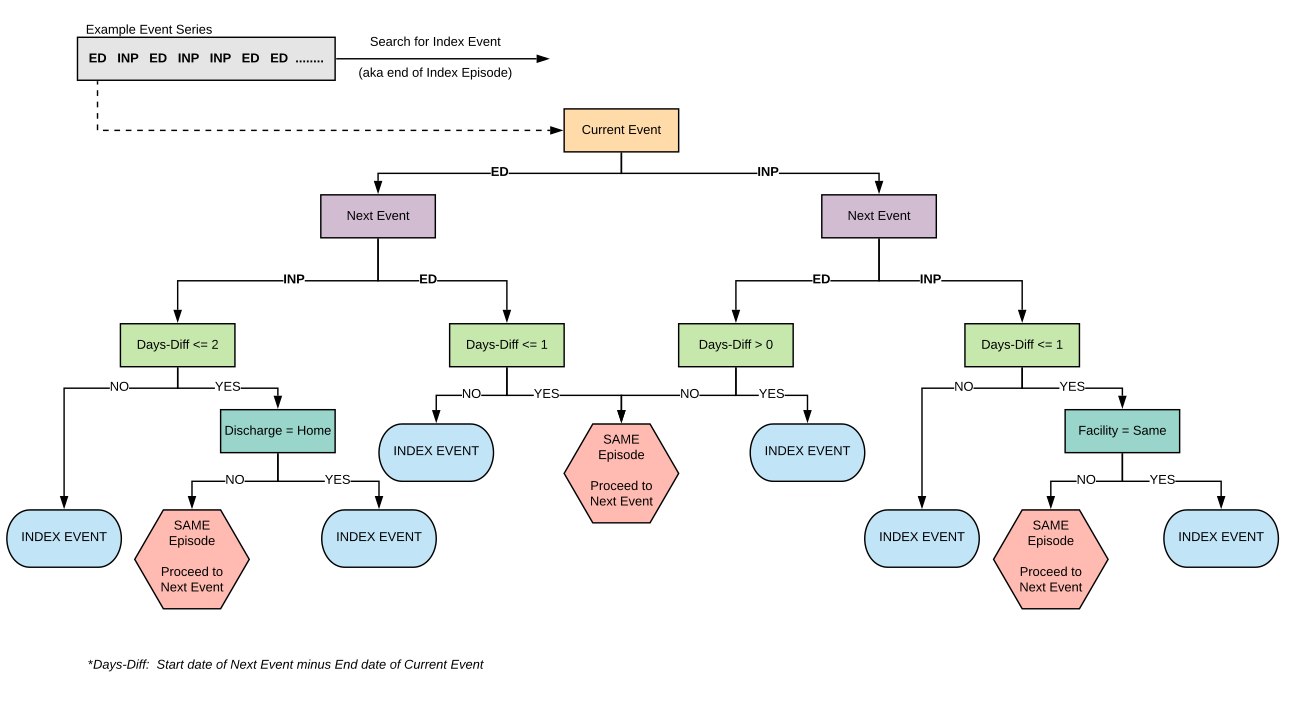
**
