# Supplementary material for: Machine Learning For Risk Prediction After Heart Failure Emergency Department Visit or Hospital Admission Using Administrative Health Data
Source: PLOS Digit Health. 2024 Oct 25;3(10):e0000636. doi: 10.1371/journal.pdig.0000636 (PMC11508085; doi:10.1371/journal.pdig.0000636)

**Supplementary Figure 2**. Flowchart describing samples sizes (index events) in the secondary analysis cohort (for the outcomes of 30-day and 1-year heart failure (HF) hospital readmission or death), used for training and for validating the models for each outcome, after applying the episode creation and study exclusion criteria.


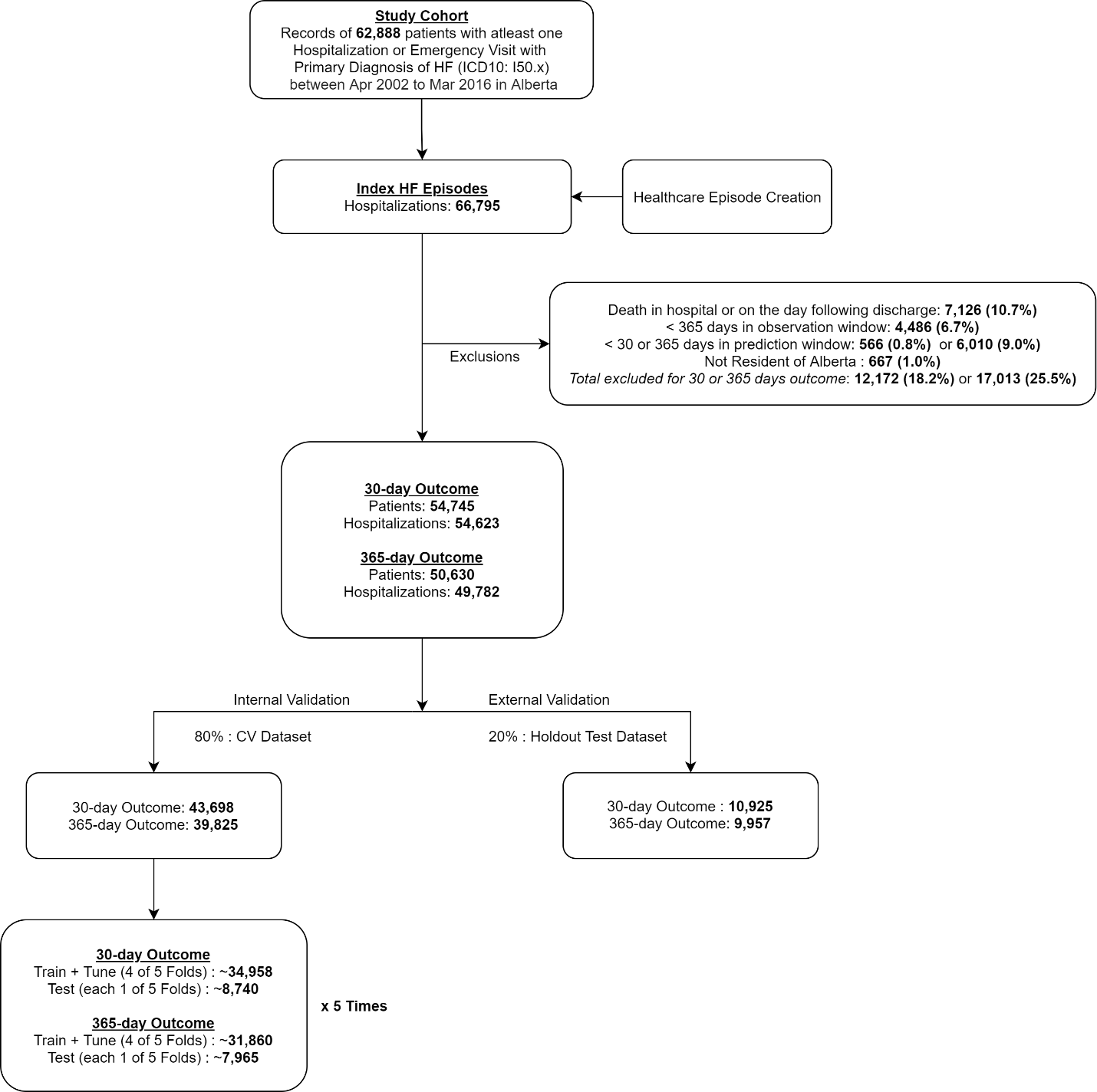

Supplement: S2 Fig — (DOCX) [file pdig.0000636.s008.docx]
