# Supplementary material for: Machine Learning For Risk Prediction After Heart Failure Emergency Department Visit or Hospital Admission Using Administrative Health Data
Source: PLOS Digit Health. 2024 Oct 25;3(10):e0000636. doi: 10.1371/journal.pdig.0000636 (PMC11508085; doi:10.1371/journal.pdig.0000636)
Supplement: S3 Fig — (DOCX) [file pdig.0000636.s009.docx]

**Supplementary Figure 3.** Feature extraction from the relational database structure of administrative health data.

**
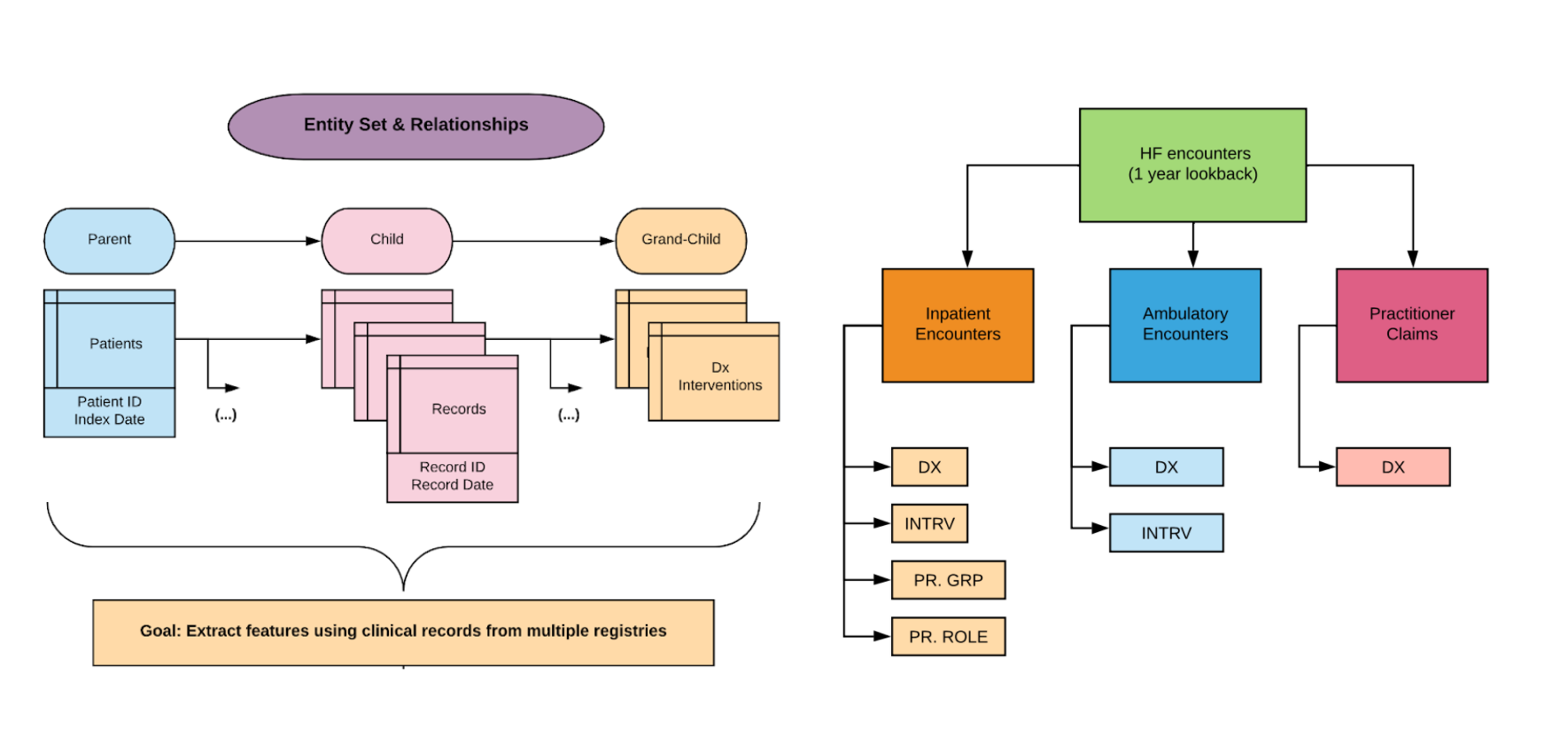
**
