# Supplementary material for: Machine Learning For Risk Prediction After Heart Failure Emergency Department Visit or Hospital Admission Using Administrative Health Data
Source: PLOS Digit Health. 2024 Oct 25;3(10):e0000636. doi: 10.1371/journal.pdig.0000636 (PMC11508085; doi:10.1371/journal.pdig.0000636)
Supplement: S4 Fig — (DOCX) [file pdig.0000636.s010.docx]

**Supplementary Figure 4.** Study outcome distributions by year.

|  | 30-day outcomes | 1-year outcomes |
| --- | --- | --- |
| Death among patients with HF ED visits or HF hospitalization | 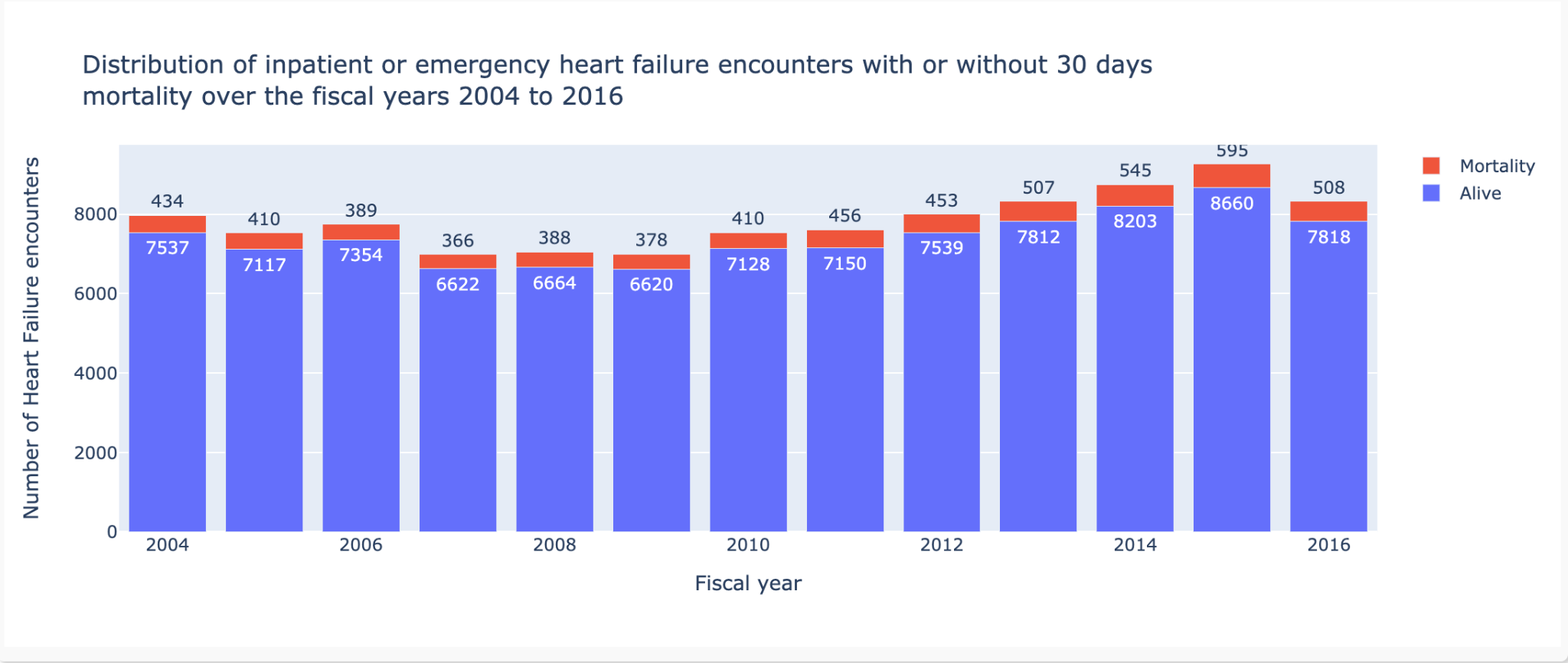 | 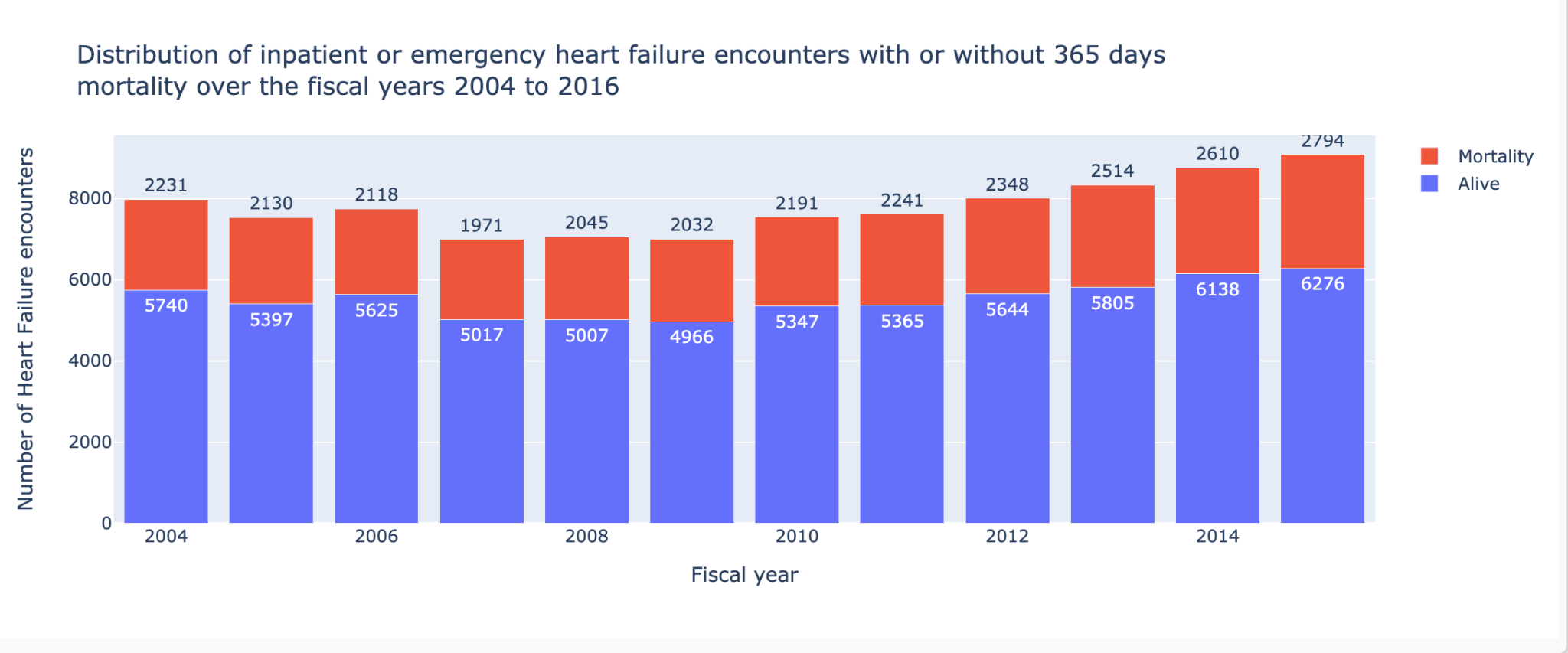 |
| HF rehospitalization or death among patients with HF ED visits or HF hospitalization | 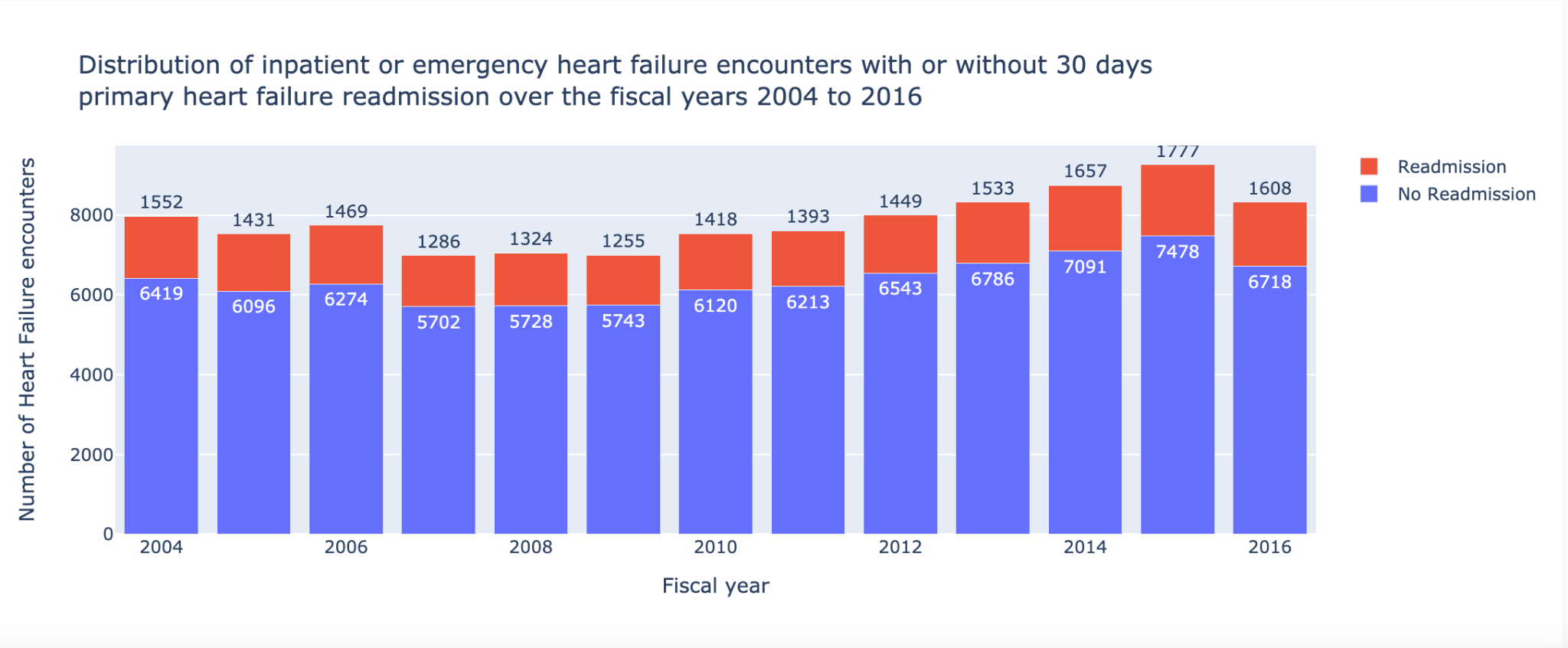 | 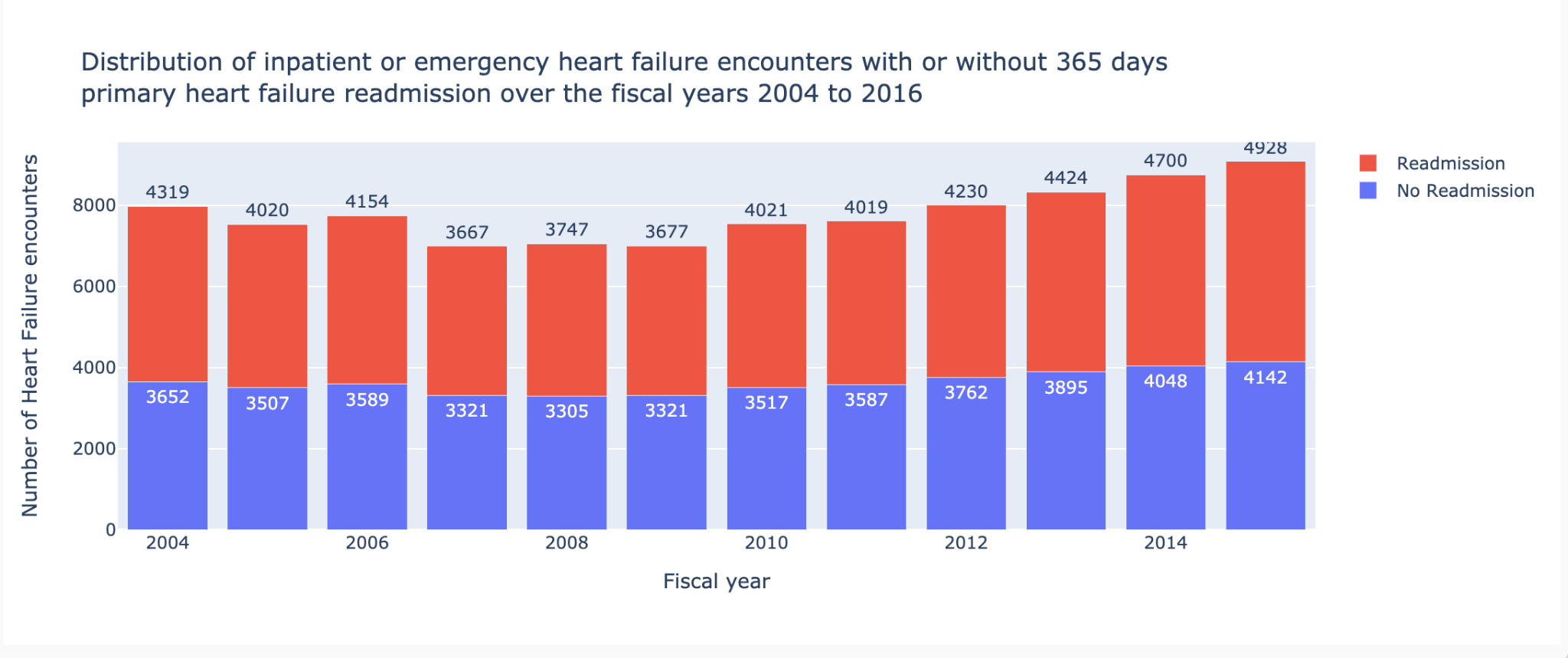 |

**Supplementary Figure 4 (continued).** Study outcome distributions by year.

|  | 30-day outcome | 365-day outcome |
| --- | --- | --- |
| Death among patients with HF hospitalization | 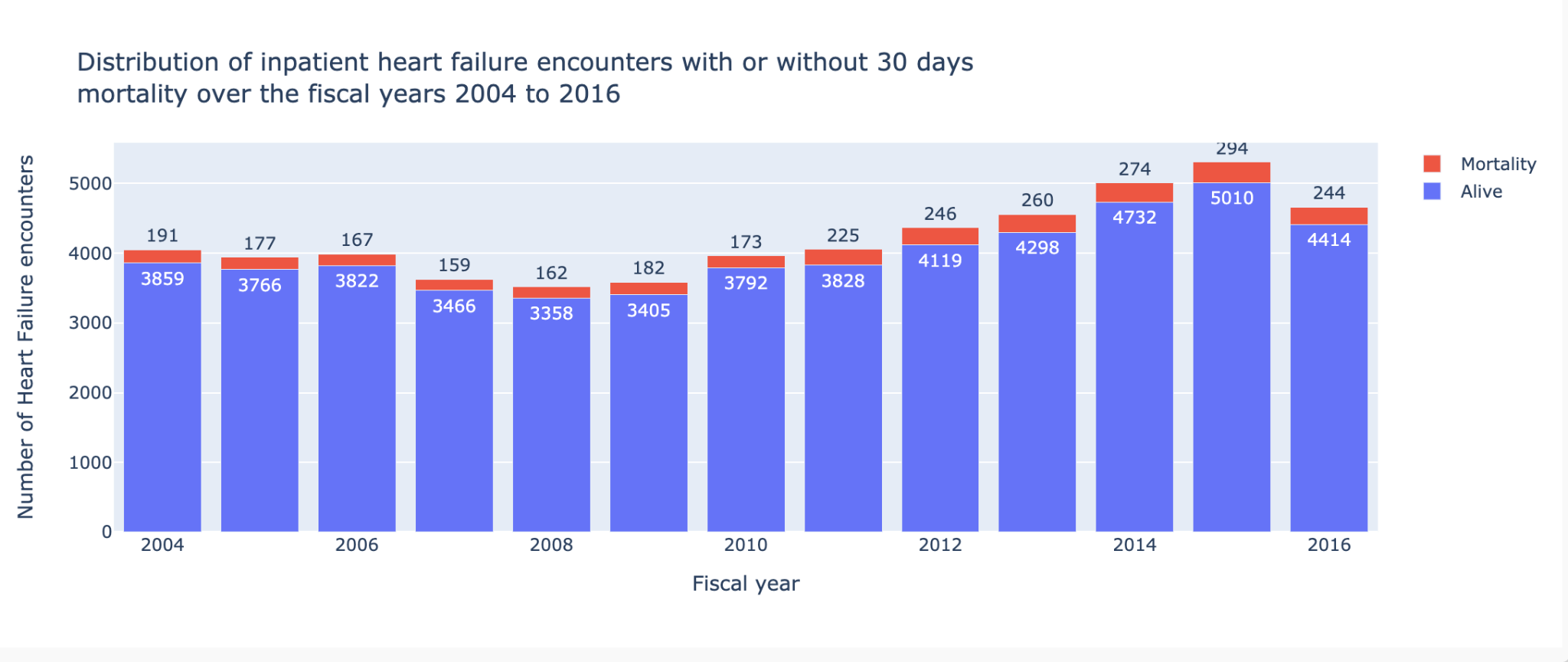 | 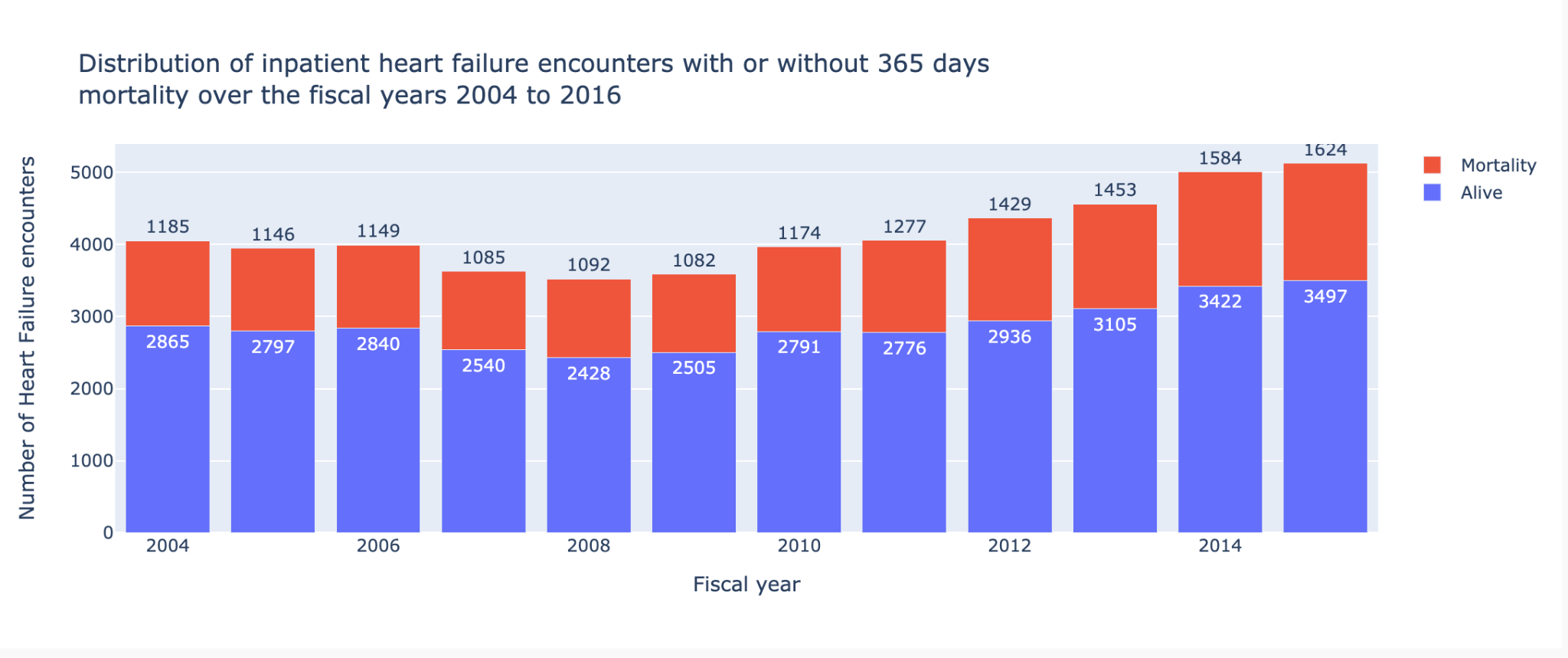 |
| HF rehospitalization or death among patient with HF hospitalization | 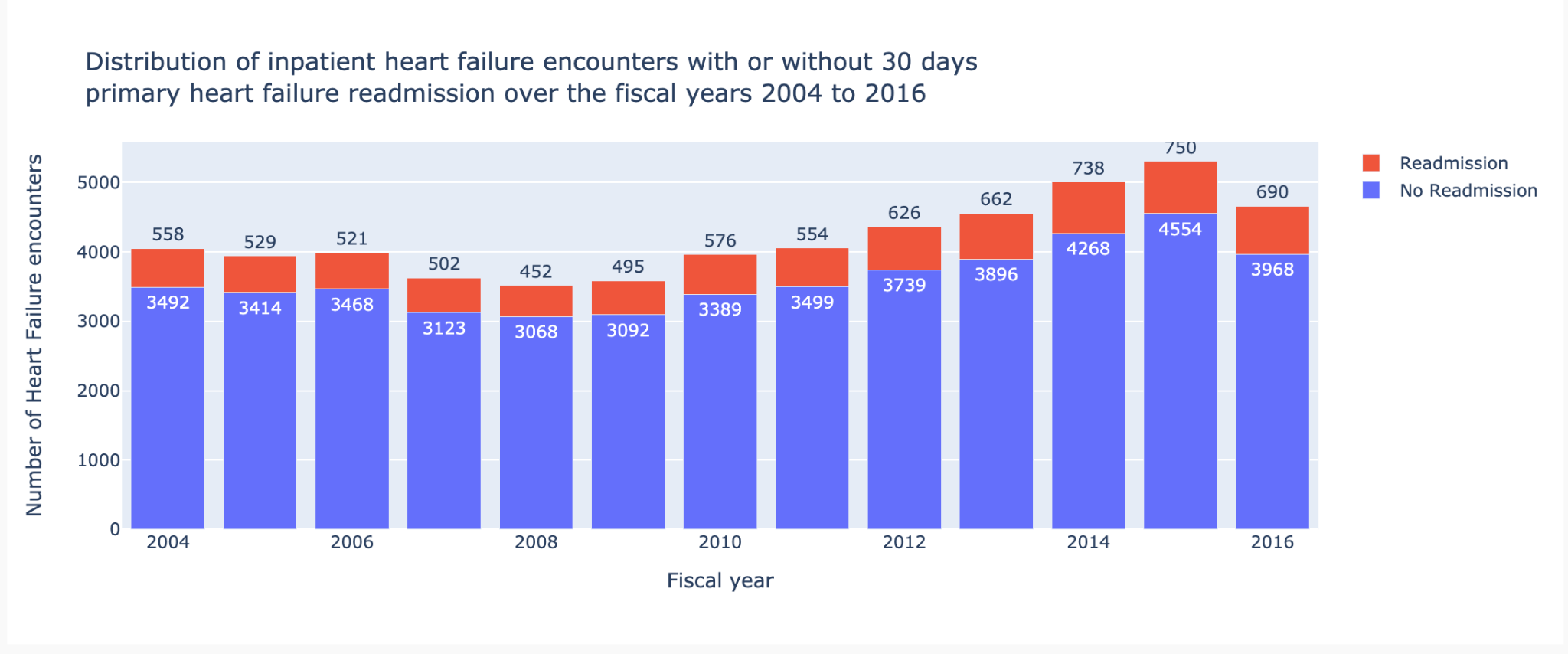 | 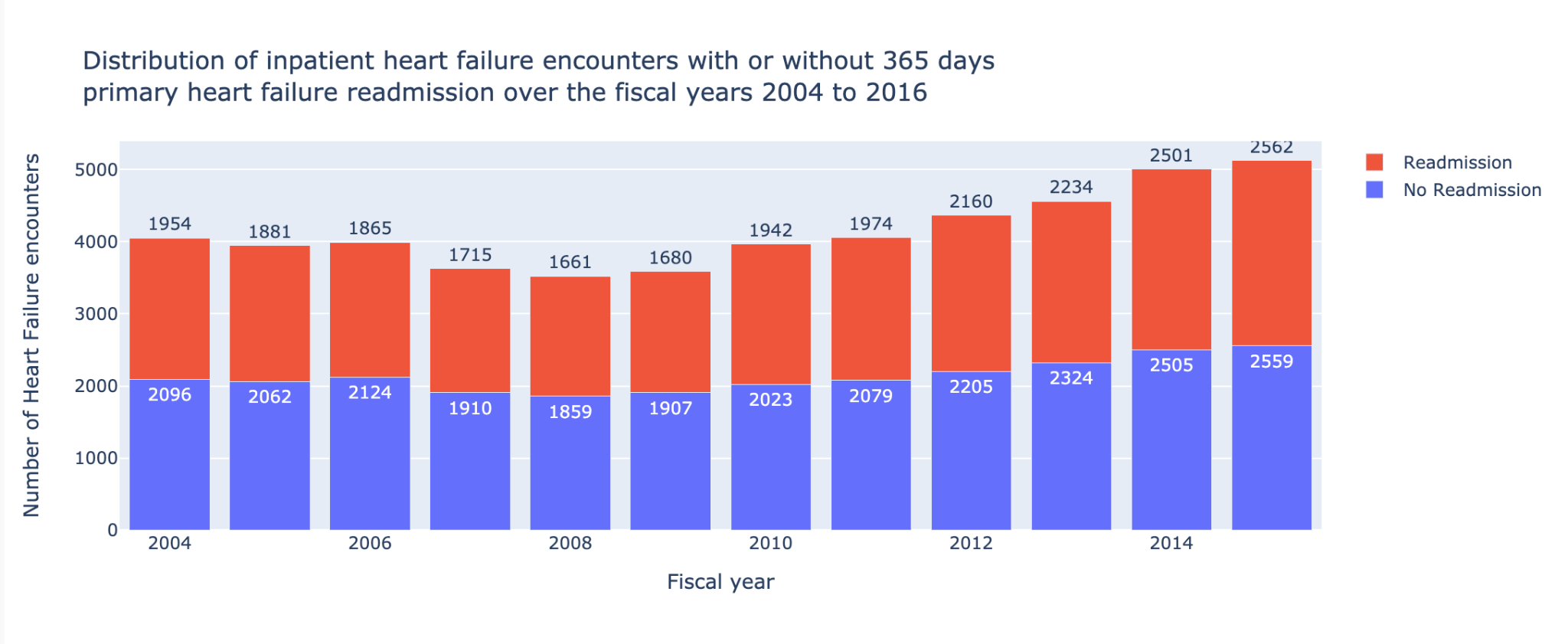 |
