# Supplementary material for: Machine Learning For Risk Prediction After Heart Failure Emergency Department Visit or Hospital Admission Using Administrative Health Data
Source: PLOS Digit Health. 2024 Oct 25;3(10):e0000636. doi: 10.1371/journal.pdig.0000636 (PMC11508085; doi:10.1371/journal.pdig.0000636)
Supplement: S5 Fig — Green to Red indicates low- to high-risk groups based on 20% quantile steps of predicted probability score. Gray bar suggests unreliable estimate due to <10 encounters in the risk group. ED-emergency department, HF-heart failure. (DOCX) [file pdig.0000636.s011.docx]

**Supplementary Figure 5.** Bar plots showing the calibration of CatBoost Models. Green to Red indicates low- to high-risk groups based on 20% quantile steps of predicted probability score. Gray bar suggests unreliable estimate due to <10 encounters in the risk group. ED-emergency department, HF-heart failure.

|  | **Distribution of predicted risk groups in external validation** | **Percent observed outcomes among predicted risk groups in external validation** |
| --- | --- | --- |
| 30-day outcome  Death among HF ED visits and HF hospitalized patients | 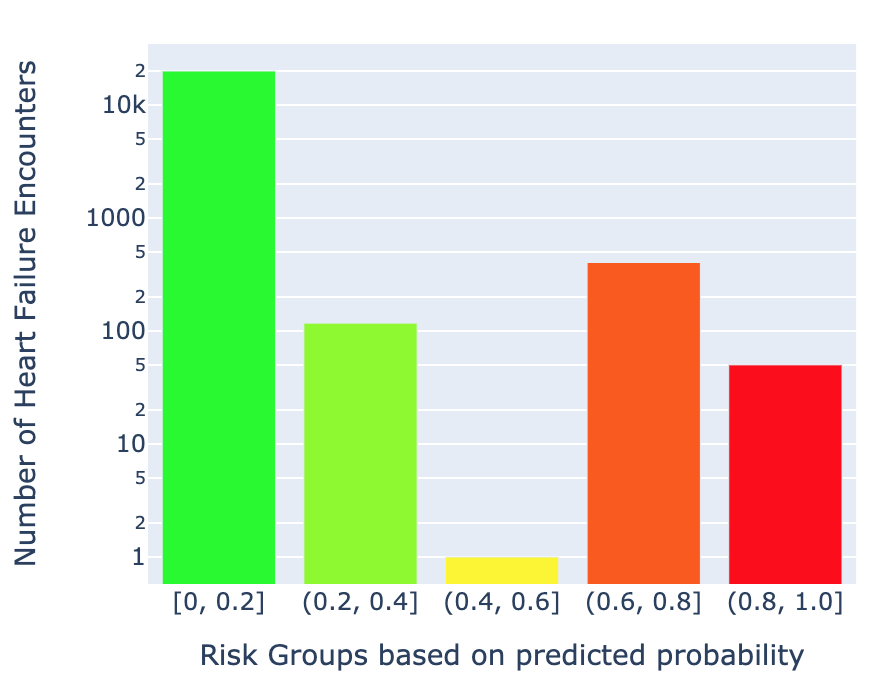 | 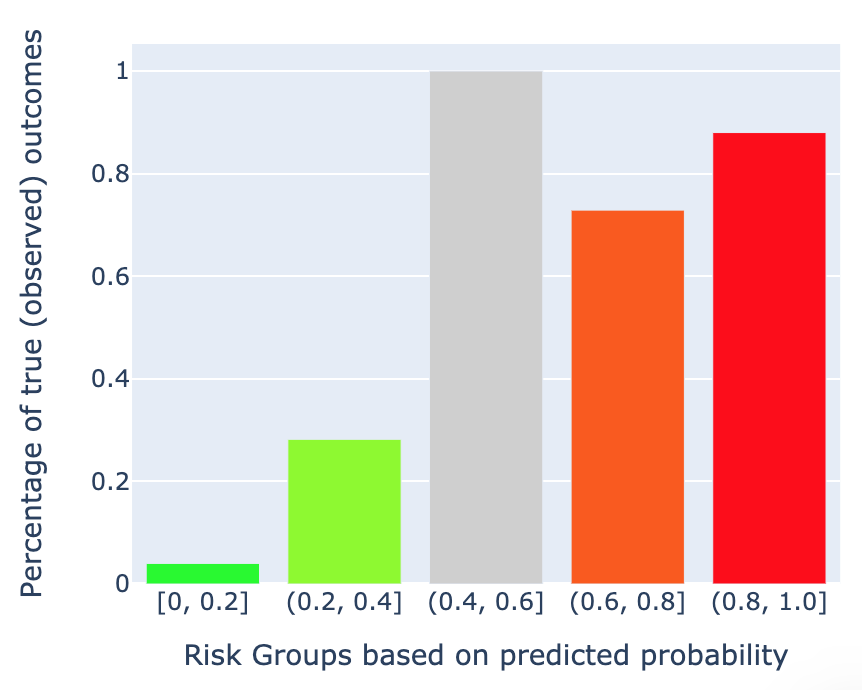 |
| 30-day outcome  HF rehospitalization or death among HF ED visits and HF hospitalized patients | 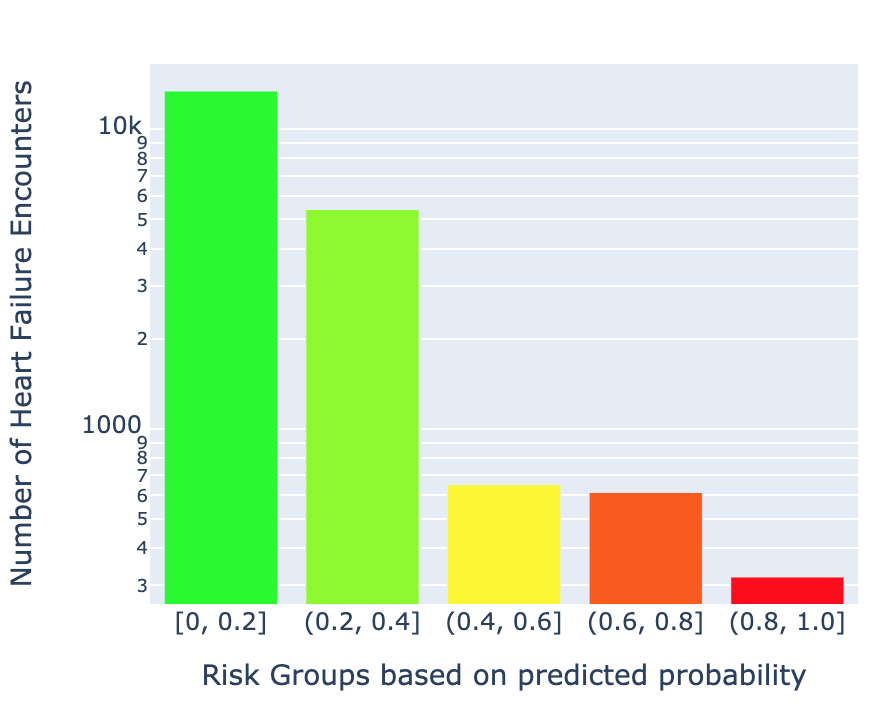 | 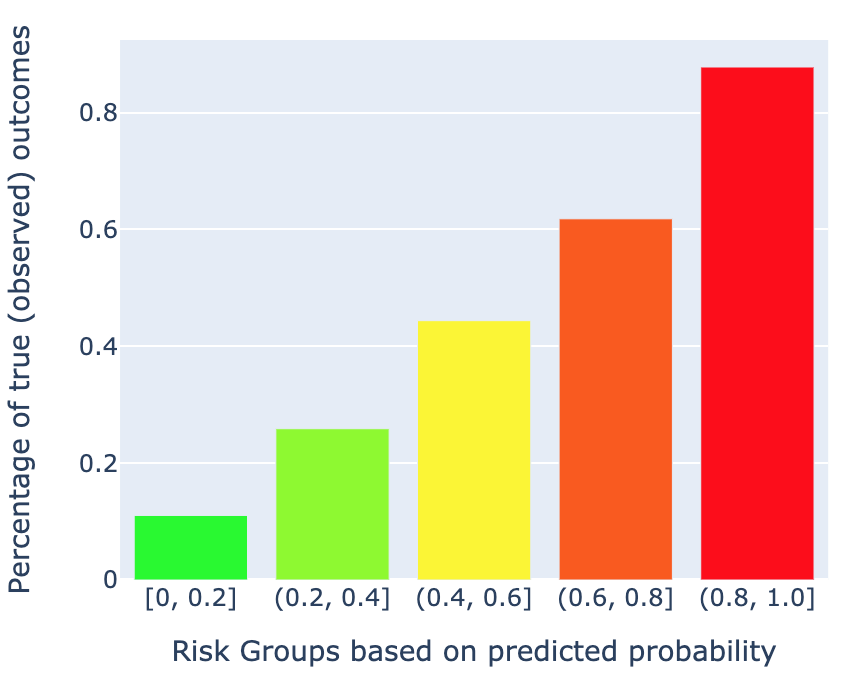 |
| 30-day outcome  HF rehospitalization or Death among HF hospitalized patients only | 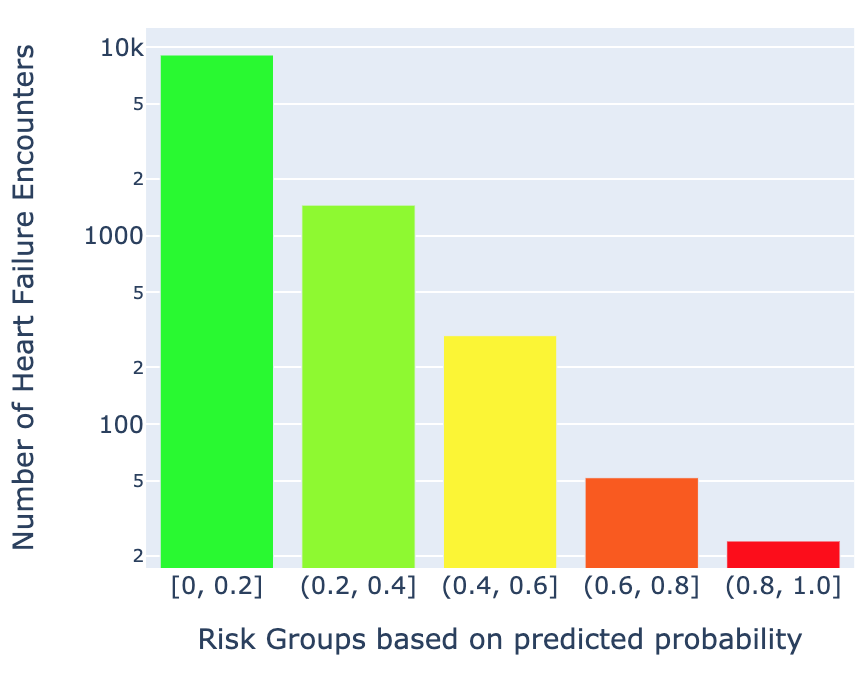 | 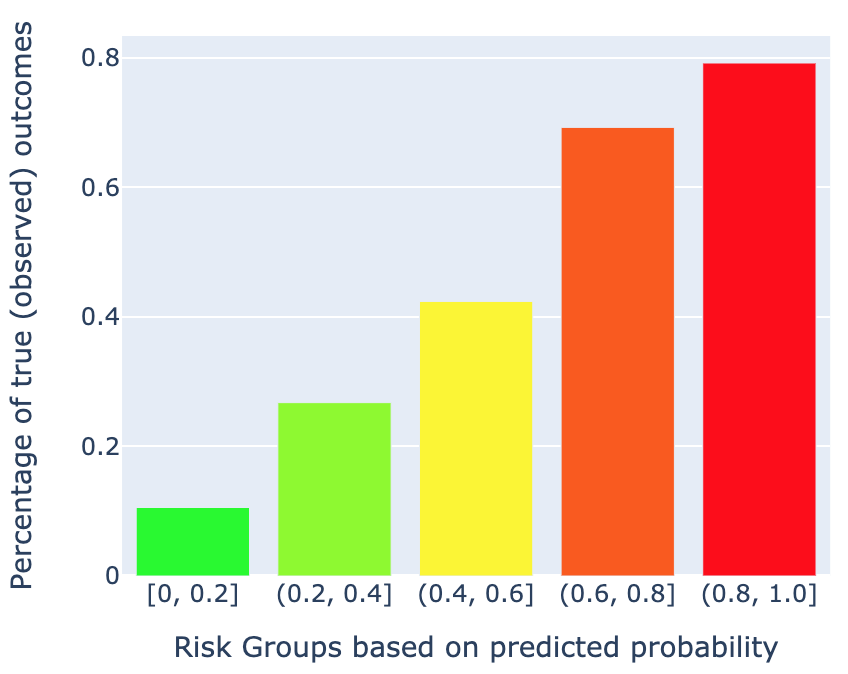 |
| 1-year outcome  death among patients with HF ED visit or HF hospitalization | 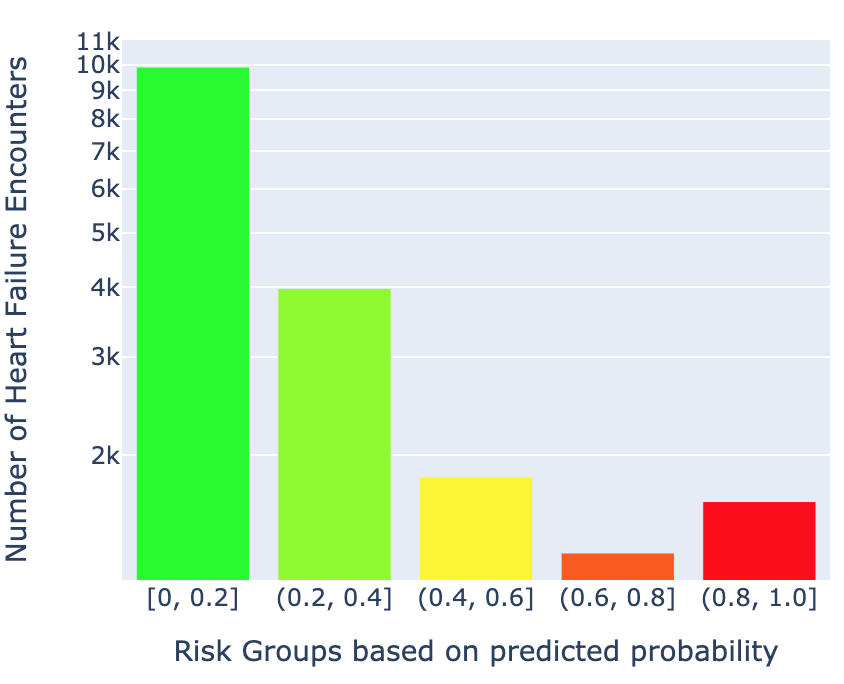 | 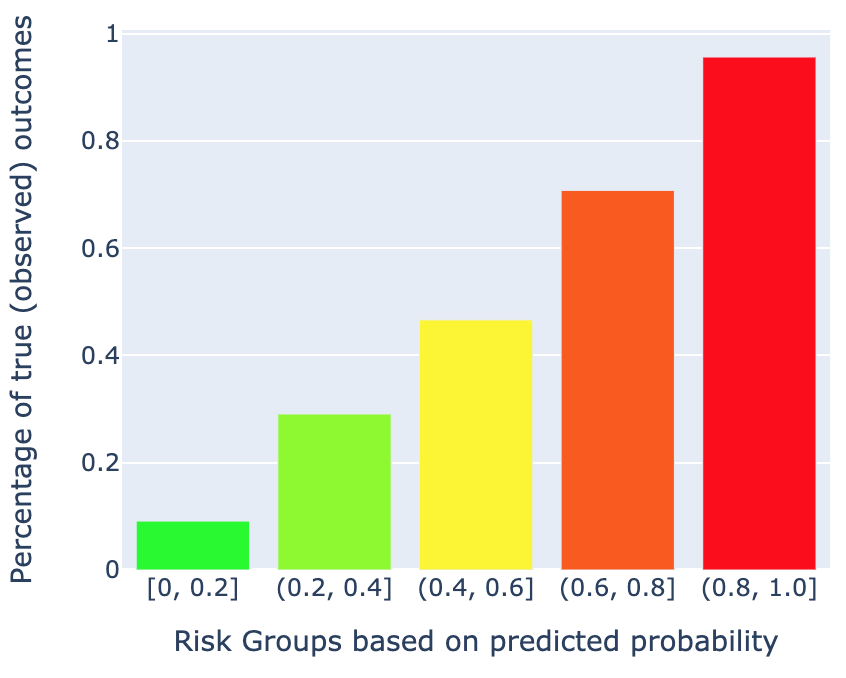 |
| 1-year outcome  HF rehospitalization or death among patients with HF ED visit or HF hospitalization | 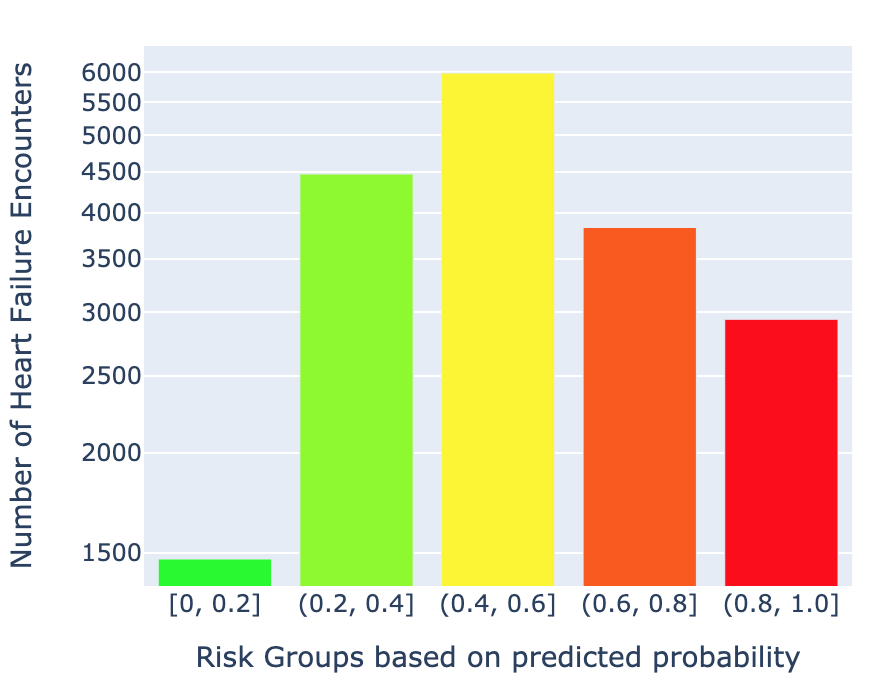 | 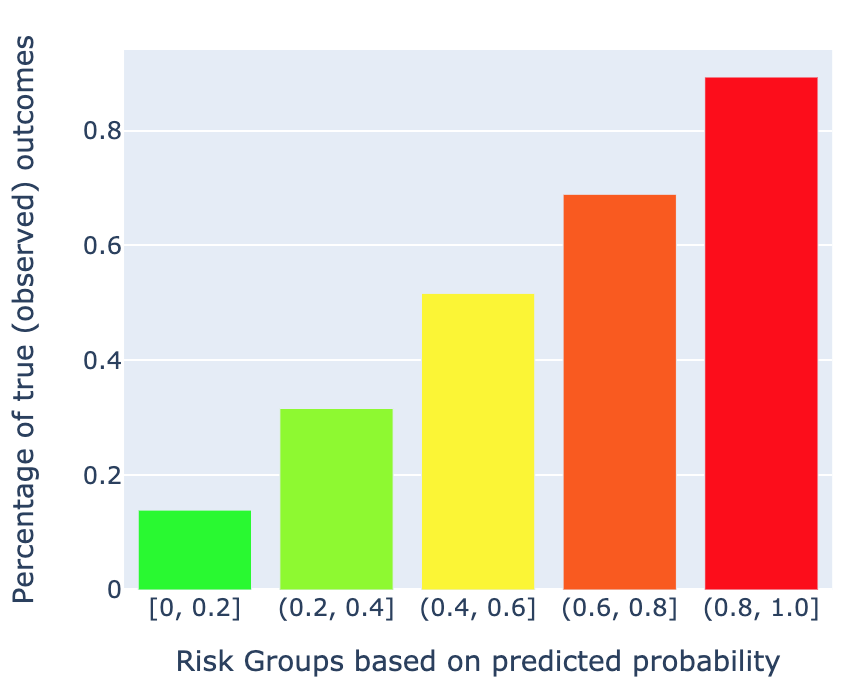 |
| 1-year outcome  HF rehospitalization or death among patients with HF hospitalization | 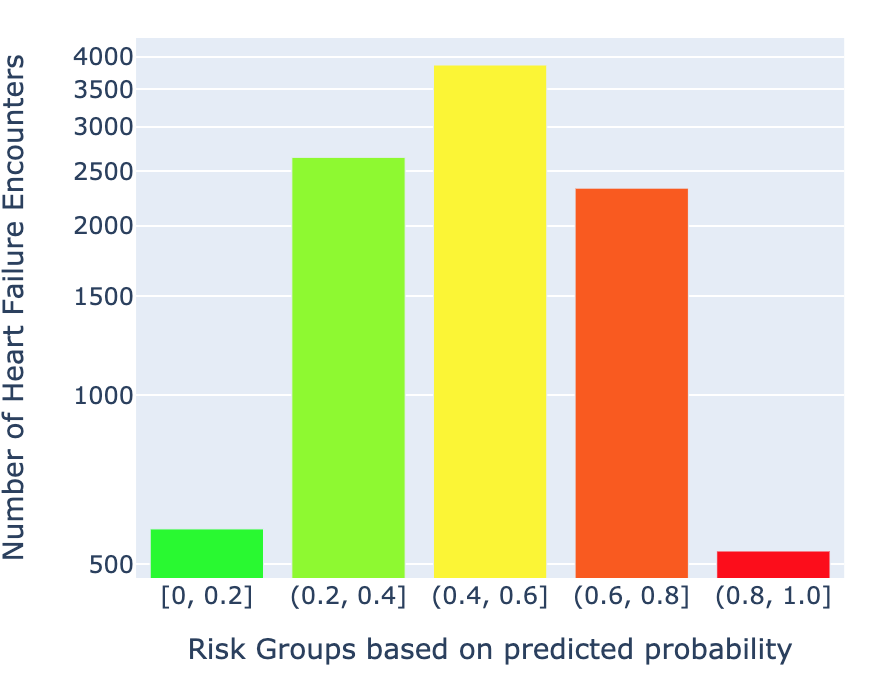 | 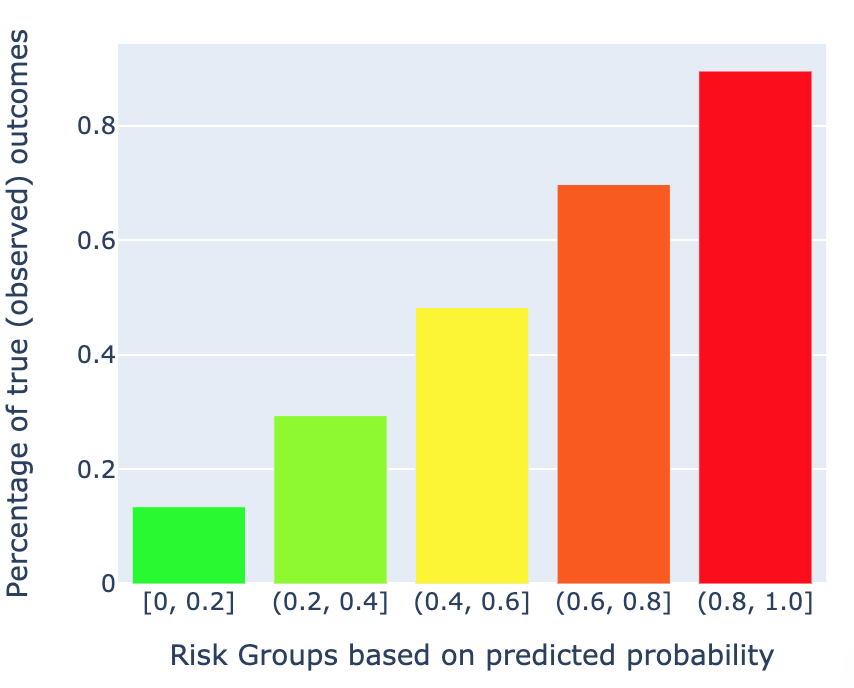 |
