# Supplementary material for: Machine Learning For Risk Prediction After Heart Failure Emergency Department Visit or Hospital Admission Using Administrative Health Data
Source: PLOS Digit Health. 2024 Oct 25;3(10):e0000636. doi: 10.1371/journal.pdig.0000636 (PMC11508085; doi:10.1371/journal.pdig.0000636)
Supplement: S6 Fig — The feature names are presented in the format “mathematical_operation(database.variable)”. For example, LAST (Hospitalizations_visits.Discharge status) refers to the discharge status assigned during a patient’s last inpatient visit. Please refer to S1 Table for the details, grouping and source database of study variables included in the analysis. (DOCX) [file pdig.0000636.s012.docx]

**Supplementary Figure 6:** Feature importance and SHAP (SHapley Additive exPlanations) analyses figures for the models presented in the paper. The feature names are presented in the format “mathematical_operation(database.variable)”. For example, LAST (Hospitalizations_visits.Discharge status) refers to the discharge status assigned during a patient's last inpatient visit. Please refer to the Supplementary Table 1 for the details, grouping and source database of study variables included in the analysis.

|  | **Catboost** | **Logistic Regression** |
| --- | --- | --- |
| **30-day HF ED visit/HF rehospitalization or death** | **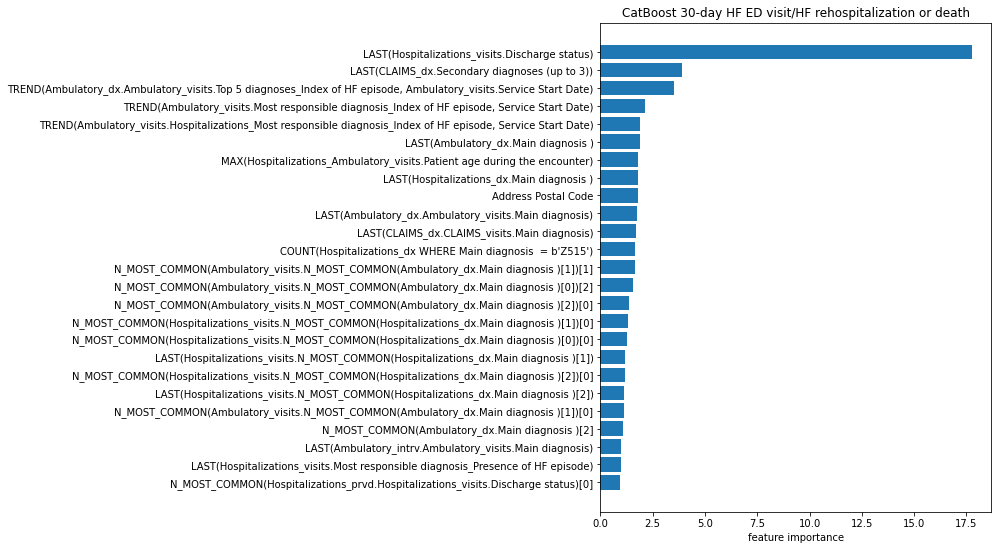** | **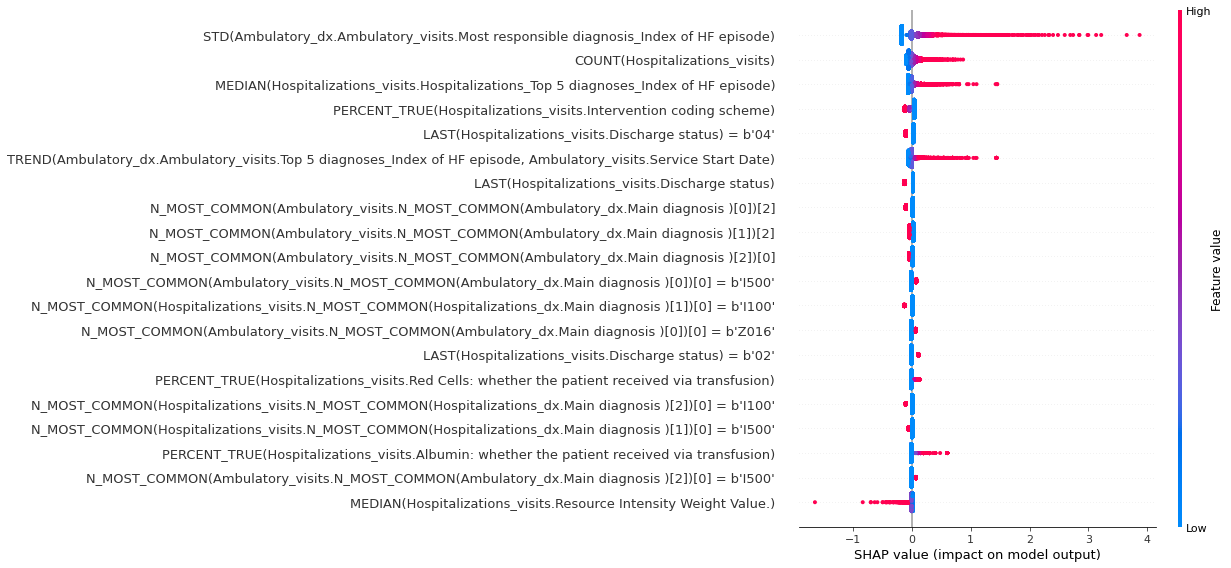** |
| **1-year HF ED visit/HF rehospitalization or death** | **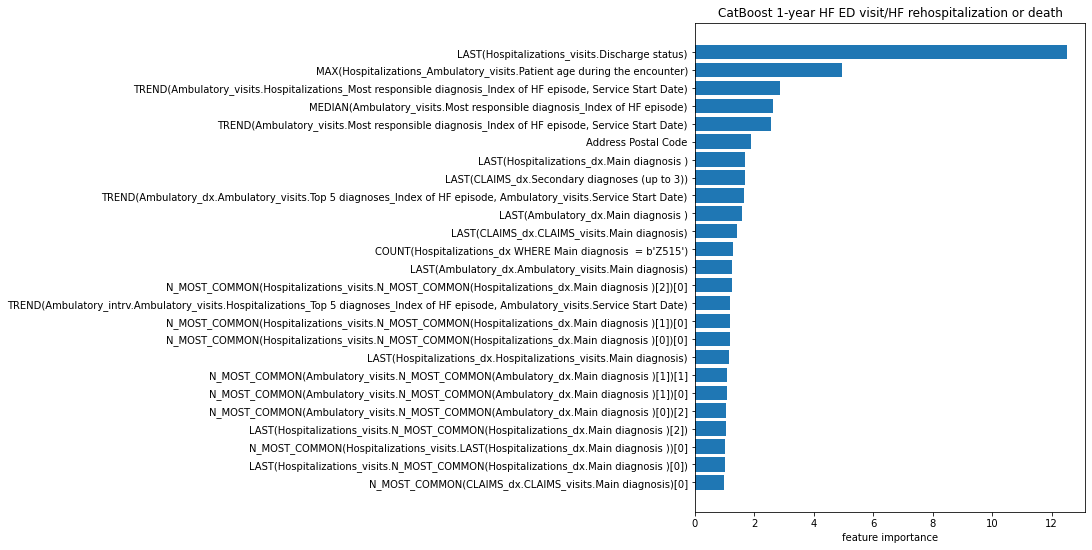** | **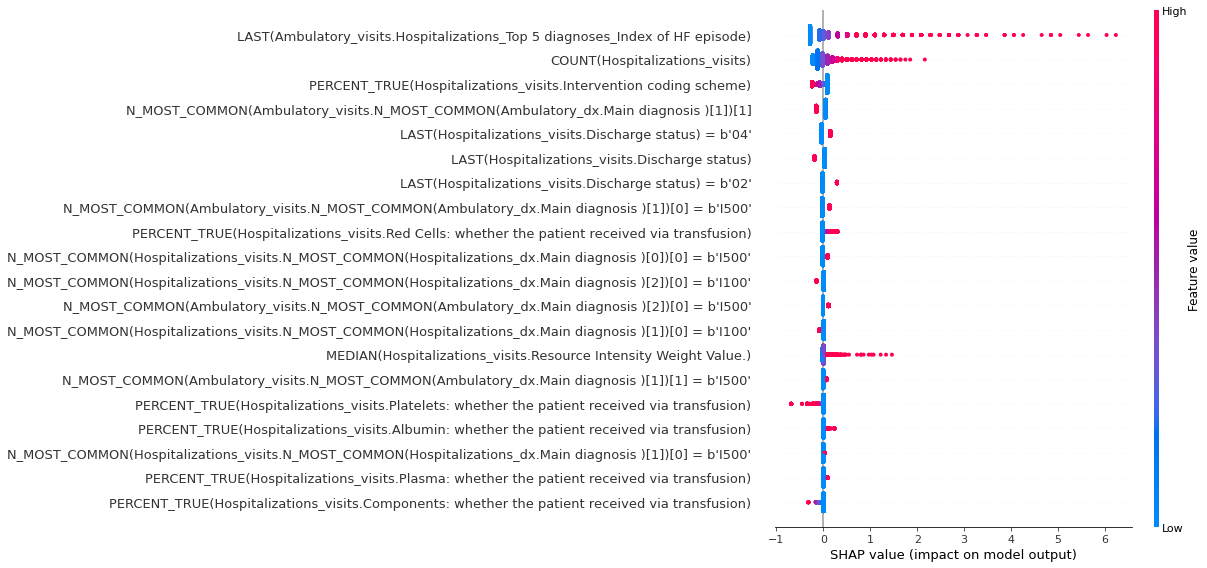** |
| **30-day Death** | **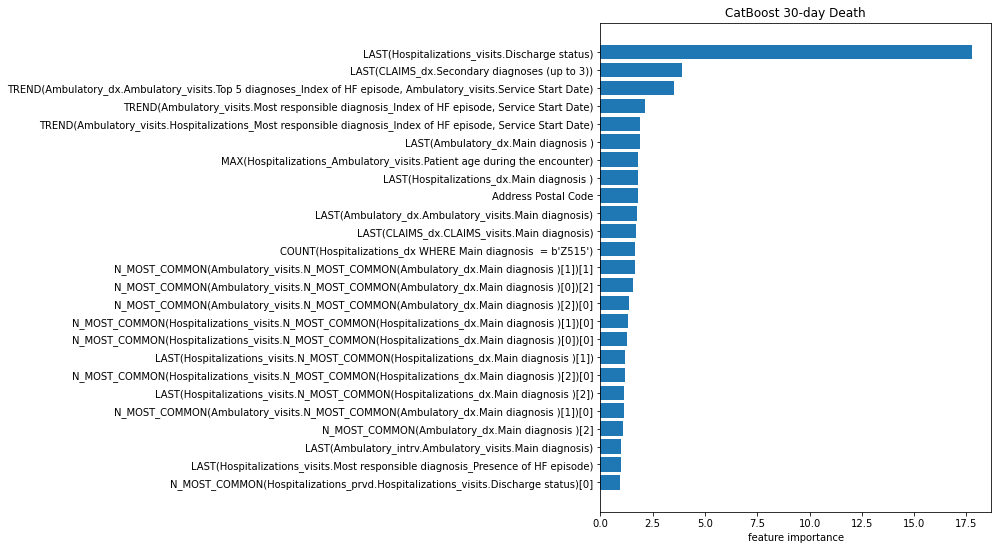** | **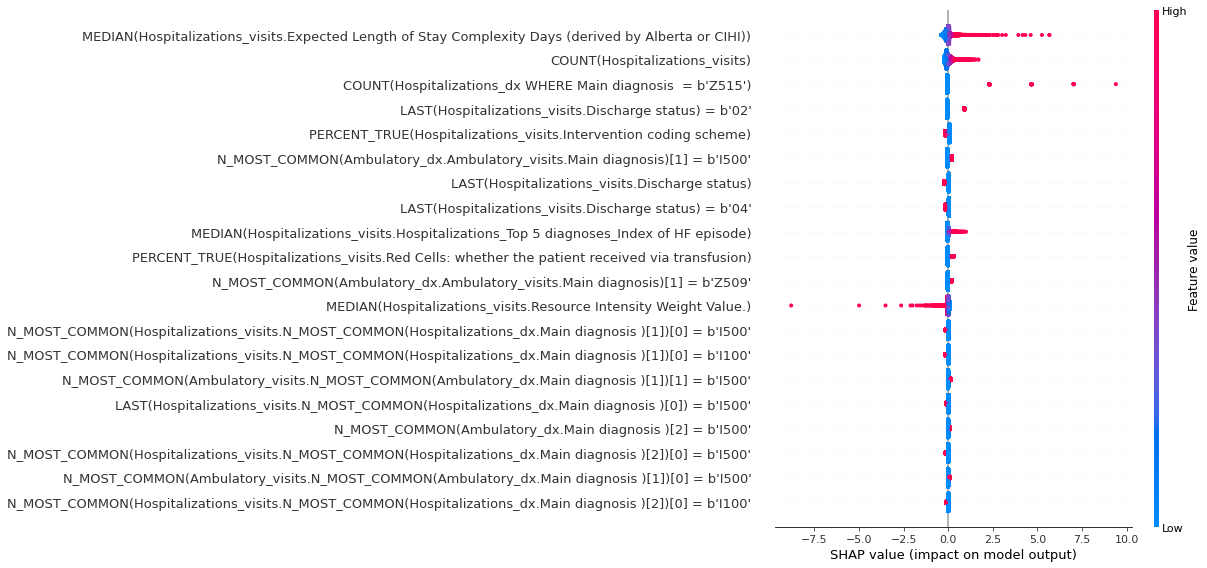** |
| **1-year Death** | **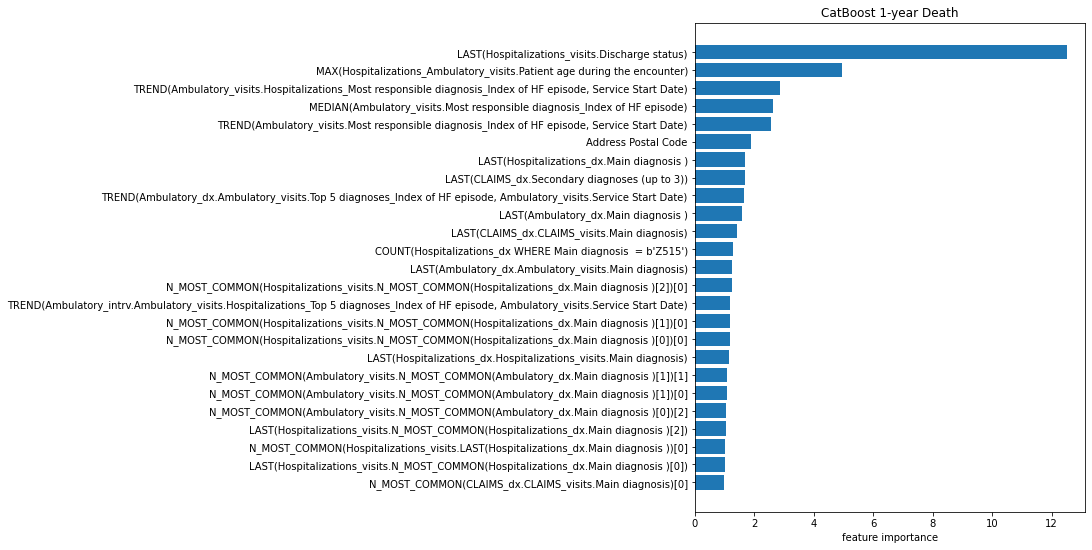** | **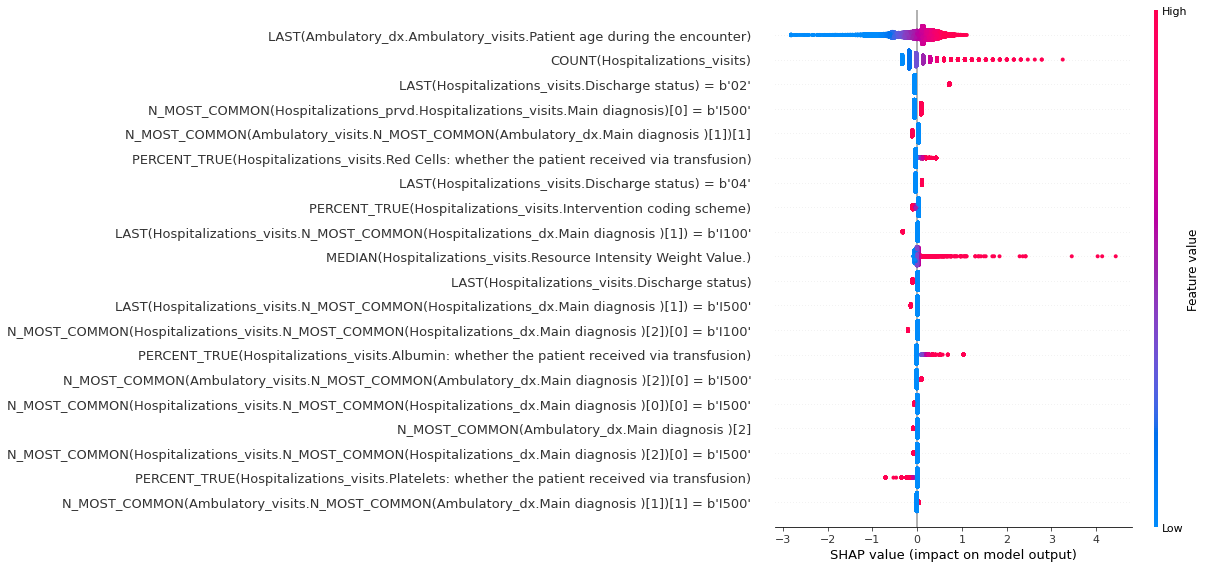** |
