# Supplementary material for: Machine Learning For Risk Prediction After Heart Failure Emergency Department Visit or Hospital Admission Using Administrative Health Data
Source: PLOS Digit Health. 2024 Oct 25;3(10):e0000636. doi: 10.1371/journal.pdig.0000636 (PMC11508085; doi:10.1371/journal.pdig.0000636)

**Supplementary Figure 7.** Bar plot representing the holdout performance in terms of AUROC scores for patient subgroups with specific heart failure etiologies and concomitant conditions.


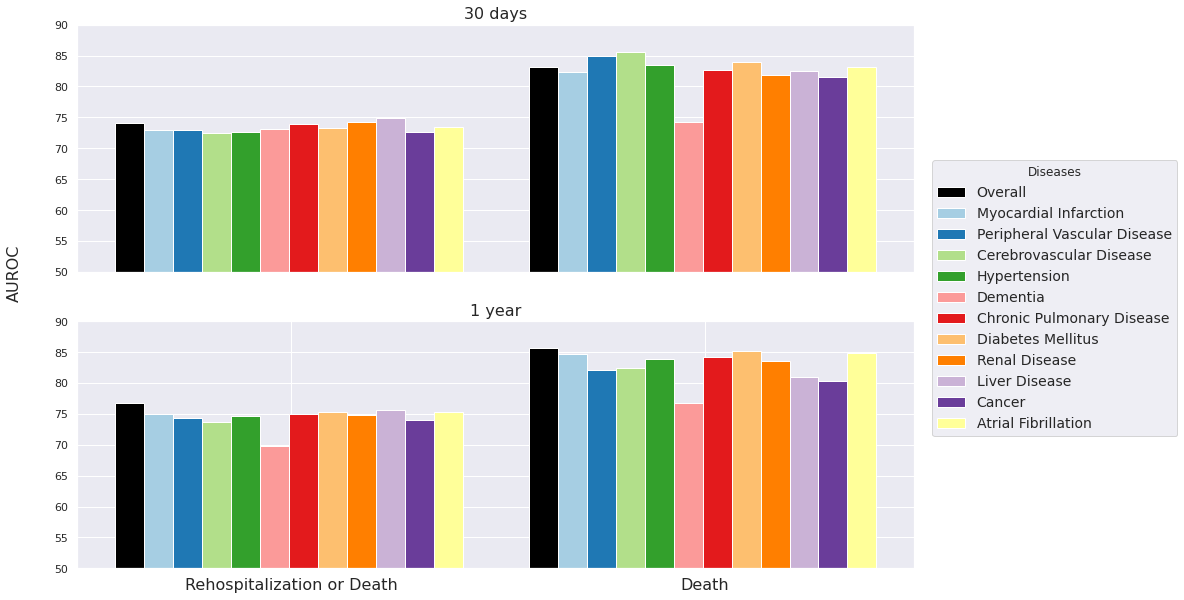

Supplement: S7 Fig — (DOCX) [file pdig.0000636.s013.docx]
